# Plasma proteomics-based brain aging signature and incident dementia risk

**DOI:** 10.1007/s11357-024-01407-6

**Published:** 2024-11-12

**Authors:** Minghao Kou, Hao Ma, Xuan Wang, Yoriko Heianza, Lu Qi

**Affiliations:** 1 Department of Epidemiology, Celia Scott Weatherhead School of Public Health and Tropical Medicine, Tulane University, New Orleans, LA USA; 2 Department of Nutrition, Harvard T.H. Chan School of Public Health, Boston, MA USA

**Keywords:** Proteomics, Aging, Dementia, Alzheimer’s disease

## Abstract

Investigating brain-enriched proteins with machine learning methods may enable a brain-specific understanding of brain aging and provide insights into the molecular mechanisms and pathological pathways of dementia. The study aims to analyze associations of brain-specific plasma proteomic aging signature with risks of incident dementia. In 45,429 dementia-free UK Biobank participants at baseline, we generated a brain-specific biological age using 63 brain-enriched plasma proteins with machine learning methods. The brain age gap was estimated, and Cox proportional hazards models were used to study the association with incident all-cause dementia, Alzheimer’s disease (AD), and vascular dementia. Per-unit increment in the brain age gap *z*-score was associated with significantly higher risks of all-cause dementia (hazard ratio [95% confidence interval], 1.67 [1.56–1.79], *P* < 0.001), AD (1.85 [1.66–2.08], *P* < 0.001), and vascular dementia (1.86 [1.55–2.24], *P* < 0.001), respectively. Notably, 2.1% of the study population exhibited extreme old brain aging defined as brain age gap *z*-score > 2, correlating with over threefold increased risks of all-cause dementia and vascular dementia (3.42 [2.25–5.20], *P* < 0.001, and 3.41 [1.05–11.13], *P* = 0.042, respectively), and fourfold increased risk of AD (4.45 [2.32–8.54], *P* < 0.001). The associations were stronger among participants with healthier lifestyle factors (all *P*-interaction < 0.05). These findings were corroborated by magnetic resonance imaging assessments showing that a higher brain age gap aligns global pathophysiology of dementia, including global and regional atrophy in gray matter, and white matter lesions (*P* < 0.001). The brain-specific proteomic age gap is a powerful biomarker of brain aging, indicative of dementia risk and neurodegeneration.

**Supplementary Information:**

The online version contains supplementary material available at 10.1007/s11357-024-01407-6.

## Introduction

Dementia is a growing public health concern, with an estimation that it will affect 131 million people globally by 2050 [1]. Alzheimer’s disease (AD) and vascular dementia are the predominant types, responsible for approximately 60 to 80% and 15% of all cases, respectively [2, 3]. Aging is the most profound risk factor for dementia [4], yet, the molecular basis for brain aging and dementia remains largely unknown.

Recent advances in large-scale omics technologies have led to the development of various “age clocks” to quantify biological aging, utilizing epigenomics, metabolomics, transcriptomics, and proteomics [5]. One such measure is the “proteomic age gap,” which refers to the difference between a person’s predicted age based on proteomics and their chronological age. A larger proteomic age gap indicates that an individual is older than expected for his/her age, which has been linked to increased risks of major diseases and mortality [6]. However, most omics-based age gaps have been limited in capturing brain-specific aging. A novel approach has recently emerged that leverages organ-enriched plasma proteome to estimate organ-specific aging, revealing significant associations of brain aging with AD prevalence and dementia progression [7]. Like the proteomic age gap, the proteomic brain age gap may provide a quantifiable way to assess deviations of biological brain aging from chronological brain aging trajectories and an important metric in understanding aging-related pathologies, including dementia.

Key questions persist regarding the associations between proteomic brain age gap and the incidence of all-cause dementia, AD, and vascular dementia, and whether these associations vary by dementia etiology. Moreover, the relationship between the proteomic brain age gap and other dementia biomarkers—such as neuroimaging indicators of brain atrophy and lesions—warrants examination. It is also crucial to investigate how lifestyle factors [8–12] and non-modifiable genetic susceptibility, most notably *APOE* polymorphism [13], might modify the associations.

In this study, we leveraged the largest proteomics dataset from the UK Biobank to estimate brain aging using brain-enriched proteins. We assessed the relationship between the proteomic brain age gap and the risk of all-cause dementia, its major subtypes, and neuroimaging measures by magnetic resonance imaging (MRI). Moreover, we examined the potential modification effects of lifestyle factors and genetic susceptibility on the associations between the brain age gap and the risk of dementia.

## Methods

### Study population

In the UK Biobank study, more than 500,000 participants were recruited from the general population at 22 assessment centers throughout the United Kingdom, aged 40–69 years between 2006 and 2010. The study population included participants with Olink proteomics assessment at baseline and those who were randomly sampled from the total UK Biobank population (*n* = 45,429). Randomized participants in the UK Biobank have been shown to be highly representative of the whole UK Biobank population [14]. Our study explored two subsets of the population, a dementia risk subset, and a brain volume subset (Supplemental Fig. 1). We included a total of 45,374 participants to investigate the associations between brain age gaps and the risk of dementia, after excluding participants with baseline dementia. For the brain volume subset, we further evaluate the associations between brain age gaps and brain volume measurements among those who attended the first wave (2014 onwards) of the MRI brain imaging study (*n* = 3695). The study was approved by the Northwest Multi-Centre Research Ethics Committee and the Tulane University (New Orleans, LA) Biomedical Committee Institutional Review Board, and written informed consent was obtained from all participants.

### Proteomics data and brain-enriched proteins

Detailed information on proteomics data and identification of brain-enriched proteins in the UK Biobank was put in Supplemental Methods. In brief, we included a total of 2916 out of 2924 proteins after excluding seven proteins (alpha-amylase 2B, cathepsin D, cathepsin S, cystatin-SN, procollagen C-endopeptidase enhancer 1, nucleophosmin, and tumor-associated calcium signal transducer 2) with over 10% missingness and one protein failing quality control (glioma pathogenesis-related protein 1). We then defined brain-enriched proteins by identifying brain-enriched annotated genes using the Gene Tissue Expression Atlas (GTEx) human tissue bulk RNA-seq database [15], following the definition proposed by the Human Protein Atlas [16]. We finally identified 127 (4.4% of 2,916) brain-enriched proteins (Supplemental Table 1).

### Proteomic brain age model and brain age gap

The method of calculating brain age gaps was adapted from a study by Argentieri et al [6], and depicted in Supplemental Fig. 2 and Supplemental Methods in detail. In brief, we trained a LightGBM machine learning model with 127 brain-enriched proteins to predict chronological age, and another model with 63 proteins after optimizing the parameters and Boruta feature selection. The 63-protein model showed a higher level of ability to explain the variation of chronological age than the 127-protein model (*R*^2^ = 0.74 vs 0.61), as well as a better correlation (0.86 vs 0.78) between predicted and chronological ages (Supplemental Table 2). SHAP (SHapley Additive exPlanations)[17] values were used to identify the relative importance of proteins in the prediction model. The predicted brain age based on 63 brain-enriched proteins was used for downstream analyses. Brain age calculation was carried out using Python v.3.6.11.

To calculate the brain age gap, we fit a local regression between predicted brain age and chronological age with a fraction parameter set to 2/3 to estimate the true population mean, due to the potential non-linearity. Brain age gaps were then calculated as the difference between the predicted brain age and the predicted population mean, and then the *z*-score was standardized for downstream analyses. After local regression, the calculated brain age gaps showed no correlation with chronological ages (*r* < 0.001).

### Assessment of incident dementia

We defined incident all-cause dementia, AD, and vascular dementia based on the *International Classification of Diseases*, *10th Revision* (ICD-10) codes: all-cause dementia (A81, F00.X, F01.X, F02.X, F03, F05.1, F10.6, G30.X, I67.3), AD (F00.X, G30.X), and vascular dementia (F01.X, I67.3). We used the dementia variables provided by UK Biobank to identify prevalent dementia, which combined information on the diagnosis of dementia through medical history and linkage to data on hospital admissions, self-reported questionnaires, and the death register data (Data Field 42018). Prevalent dementia was also defined as if the date of all-cause dementia was prior to the date of baseline assessment. Follow-up time was calculated from the date of baseline assessment to the date of dementia diagnosis, death, or the censoring date (December 31, 2022), whichever occurred first. Death and death dates were obtained by reviewing death certificates held by the *National Health Service*.

### Neuroimaging measurements

Our study made use of imaging-derived phenotypes generated by an image-processing pipeline developed and run on behalf of the UK Biobank [18, 19]. All brain MRI data were acquired using a 3 Tesla Siemens Skyra scanner and a standard Siemens 32-channel head coil and underwent standardized processing and quality control procedures. In this study, we included the volumes (in cubic millimeters) of the whole brain, white matter, gray matter, peripheral cortical gray matter, hippocampus (left and right), gray matter in the hippocampus (left and right), ventricular cerebrospinal fluid, and white matter hyperintensity. These neuroimaging biomarkers were clinically used to indicate brain atrophy and lesions and evaluate suspected dementia [4, 13]. The volume of white matter hyperintensities was derived from a T2-weighted brain MRI, and the others were from a T1 structural brain MRI. Neuroimaging measurements were corrected for image-related potential confounders, including head size (based on the volumetric scaling from the T1 head image to the standard atlas), head position (*x*, *y*, and *z* brain center of gravity and table position), and imaging center, according to published guidelines [20]. The scaled residuals by regressing neuroimaging measurements on image-related confounders were used as the outcomes in the neuroimaging analysis. The volume for white matter hyperintensity was log-transformed before regression due to skewed distribution.

### Assessment of covariates

The covariates included chronological age, sex, race, education levels, Townsend deprivation index, body mass index (BMI), smoking status, moderate alcohol consumption, physical activity, healthy sleep patterns, healthy diet, social isolation, depression, hearing problems, hypertension, type 2 diabetes, high cholesterol, traumatic brain injury, history of cardiovascular diseases, air pollution (nitrogen dioxide [NO_2_]; particulate matter with diameters of ≤ 2.5 µm [PM_2.5_]), and eye problems, following the latest update of dementia risk factors [21]. For neuroimaging analyses, we further included other baseline neurodegenerative processes, such as Parkinson’s disease and multiple sclerosis. Detailed information about the assessment of covariates was described in the Supplemental Material (Supplemental Methods, Supplemental Table 3). We handled missing covariates with the missing indicators for categorical variables and mean imputation for continuous variables.

### Genetic susceptibility

The genetic susceptibility of dementia was represented by the polygenetic risk score (PRS) for AD and *APOE* ε4 allele. The PRS for AD was generated using a Bayesian approach applied to meta-analyzed summary statistics GWAS data entirely from external GWAS data and released by Thompson et al. [22]. This PRS was then categorized into low (the lowest tertile), intermediate (the middle tertile), and high (the highest tertile) risk. The number of *APOE* ε4 alleles was determined by two *APOE* SNPs, rs7412 and rs429358. The number of *APOE* ε4 alleles was coded as 0 (ε2/ε2, ε2/ε3, ε3/ε3), 1 (ε3/ε4, ε2/ε4), and 2 (ε4/ε4), respectively [23]. SNP genotyping, imputation, and quality control of the genetic data were performed by the UK Biobank team [24].

### Statistical analysis

For brain age gaps, we first defined participants with *z*-score <  − 2 or > 2 as extreme agers (extremely young or extremely old) [7] and then classified the remaining participants into four evenly groups. Baseline characteristics were then summarized by these groups of participants. We also compared baseline characteristics using the ANOVA *f*-test for continuous variables and the Chi-square test for categorical variables. To evaluate the robustness of the model in different population subgroups, we assessed model performance by examining key metrics, such as Pearson’s correlation, *R*-squared (*R*^2^), and root mean squared error (RMSE) in subgroups of baseline characteristics.

Cox proportional hazards regression was used to quantify the associations between the brain age gap *z*-score and risk of all-cause dementia, AD, and vascular dementia. To validate the proportional hazards assumption, we used Schoenfeld residuals to check whether the hazard ratios were time-dependent by the brain age gap. Brain age gaps were modeled continuously (per unit increment of *z*-score) and categorically (extreme agers vs non-extreme agers). In the basic model (Model 1), we adjusted for chronological age and sex. In Model 2, we further adjusted for race (Whites or non-Whites), education levels (low, medium, or high), Townsend deprivation index (< median or ≥ median), body mass index (BMI), smoking status (never smokers or ever smokers), alcohol consumption (moderate alcohol consumption or not), physical activity (active or inactive), healthy sleep patterns (yes or no), healthy diet (yes or no), social isolation (yes or no), depression (yes or no), hearing problems (yes or no), hypertension (yes or no), type 2 diabetes (yes or no), high cholesterol (yes or no), traumatic brain injury (yes or no), history of CVD (yes or no), NO_2_, and PM_2.5_. To adjust the potential confounding from genetic susceptibility of dementia, we further adjusted the number of *APOE* ε4 allele, and the PRS for AD based on Model 2, respectively (Models 3 and 4). The model with the most conservative HRs was considered the fully adjusted model. The potential nonlinearity between brain age gaps and risk of dementia was evaluated using a restricted cubic spline with 5 knots at the 5th, 27.5th, 50th, 72.5th, and 95th percentiles of the brain age gap *z*-score, with 0 set as the reference point.

Several subgroup analyses were then conducted by modifiable lifestyle factors (smoking, alcohol use, physical activity, diet, and sleep pattern) and genetic susceptibility of dementia (the number of *APOE* ε4 alleles, and the PRS for AD) in the fully adjusted model. The interaction was tested using the Wald test by adding the interaction terms with brain age gap *z*-scores into the model. We conducted several sensitivity analyses to demonstrate the robustness of our findings. First, we repeated the analyses with the 127-protein brain age gap before feature selection to compare its performance with the 63-protein brain age gap in relation to dementia risk. Second, we applied the Fine and Gray method to control the potential competing risk of death in the abovementioned models. Third, we conducted a complete case analysis after excluding participants with missing covariates. Fourth, eye problems were further adjusted as a sensitivity analysis since their significant missingness in the study population (15,609 out of 45,374 with self-reported vision problems). Fifth, given the UK Biobank population is younger and healthier than the general population with a low incidence of dementia, we further tested the associations among participants ≥ 60 years old (20,408 out of 45,374 included). Finally, we assessed the associations after excluding the first 5 years of follow-up to evaluate potential reverse causality (44,149 out of 45,374 included).

Generalized linear regression models were fitted to quantify the associations between brain age gaps and neuroimaging measurements. The time interval between baseline and MRI imaging assessment and prevalent Parkinson’s disease and multiple sclerosis were adjusted in addition to the fully adjusted model in the main analysis.

All statistical analyses, unless otherwise stated, were done with SAS version 9.4 (SAS Institute Inc. Cary, NC, USA). All figures were created using RStudio version 4.2.2. A two-tailed *P*-value of < 0.05 was considered statistically significant.

### Role of the funding source

The funders of the study had no role in study design, data collection, data analysis, data interpretation, or writing of the report.

## Results

### Study population and proteomic brain age

The baseline characteristics of 45,374 participants are displayed in Supplement Table 4. Of the 45,374 participants, 2.6%, 95.2%, and 2.1% had brain age gap *z*-scores <  − 2, between − 2 and 2, and > 2, respectively, and were defined as extreme young agers, non-extreme agers, and extreme old agers. Among non-extreme agers (Table 1), participants with higher brain age gaps tended to be older, women, and White. Participants with higher brain age gaps were less likely to have favorable socioeconomic status (education levels and Townsend deprivation index) and lifestyles (e.g., ever smoking, healthy diet, physical activity, sleep pattern). They were also more likely to have prevalent health conditions (hypertension, type 2 diabetes, high cholesterol, hearing problems, and CVD), and psychological problems (social isolation and depression). All the abovementioned differences reached statistical significance in tests (*P* < 0.05), while prevalent traumatic brain injury, exposure to air pollution, and genetic susceptibility of dementia (*APOE* ε4 carriers, and high PRS for AD) were similar across groups of participants (*P* > 0.05).

**Table 1:** Baseline characteristics of the study participants by brain age gap after excluding extreme agers

Baseline characteristics	Total (*n* = 43,208)	Quartile 1 (*n* = 10,802)	Quartile 2 (*n* = 10,802)	Quartile 3 (*n* = 10,802)	Quartile 4 (*n* = 10,802)	*P*-value^a^
Chronological age, years	57.0 (8.19)	55.4 (8.26)	57.1 (8.46)	58.4 (8.04)	57.1 (7.70)	< 0.001
Men, %	45.8	48.4	46.6	44.9	43.5	< 0.001
White, %	93.6	92.7	93.1	94.6	94.0	< 0.001
Body mass index, kg/m^2^	27.5 (4.81)	27.2 (4.58)	27.4 (4.73)	27.5 (4.78)	27.8 (5.11)	< 0.001
Education level						< 0.001
Low, %	35.0	30.7	34.3	37.5	37.4	
Medium, %	17.6	17.5	17.9	17.5	17.5	
High, %	47.4	51.8	47.8	45.0	45.2	
Townsend deprivation index	− 1.2 (3.19)	− 1.3 (3.12)	− 1.2 (3.17)	− 1.2 (3.16)	− 1.0 (3.27)	< 0.001
Never smoking, %	54.1	58.0	54.7	52.3	51.2	< 0.001
Moderate alcohol consumption, %	52.7	52.0	52.8	52.4	53.4	0.251
Physical active, %	58.6	60.3	60.2	58.4	55.3	< 0.001
Healthy diet, %	36.8	37.0	38.1	36.7	35.5	0.001
Healthy sleep pattern, %	37.0	39.1	37.4	36.5	35.0	< 0.001
Hypertension, %	56.3	50.7	56.1	59.6	58.9	< 0.001
Type 2 diabetes, %	3.2	1.7	2.7	3.8	4.5	< 0.001
High cholesterol, %	20.0	15.6	19.2	22.9	22.5	< 0.001
Social isolation, %	14.3	13.1	13.7	14.5	16.0	< 0.001
Depression, %	24.1	23.1	22.9	23.6	26.7	< 0.001
Hearing problems, %	25.8	24.7	25.9	27.2	25.4	0.001
CVD history, %	9.0	6.4	8.5	10.0	11.1	< 0.001
Traumatic brain injury, %	0.1	0.0	0.1	0.0	0.1	0.506
NO_2_, µg/m^3^	29.4 (9.31)	29.3 (9.33)	29.4 (9.32)	29.3 (9.28)	29.5 (9.29)	0.578
PM_2.5_, µg/m^3^	10.0 (1.07)	10.0 (1.07)	10.0 (1.06)	10.0 (1.07)	10.0 (1.06)	0.117
Number of the APOE e4 alleles						0.173
0, %	70.8	70.6	70.8	71.1	70.8	
1, %	26.2	26.8	26.1	25.8	26.1	
2, %	3.0	2.6	3.1	3.1	3.0	
PRS for Alzheimer’s disease						0.792
Low, %	33.0	32.9	33.0	33.0	33.1	
Medium, %	33.2	33.4	33.4	33.4	32.6	
High, %	33.8	33.6	33.6	33.6	34.3	

*CVD* cardiovascular disease, *NO*_*2*_ nitrogen dioxide, *PM*_*2.5*_ particulate matter with diameters of ≤ 2.5 µm, *PRS* polygenic risk score

^a^*P*-values were tested by ANOVA *f*-test for continuous variables and Chi-Square test for categorical variables

The model performance was robust across various population subgroups, as shown in Supplemental Table 5. The RMSE across subgroups ranged from 4.04 to 4.62 years, and the *R*^2^ values varied between 0.60 and 0.75, indicating that the model consistently predicts brain age across groups with minimal bias. Strong linear relationships between predicted brain ages and chronological ages were observed across subgroups, with Pearson’s correlation coefficients ranging from 0.77 to 0.86. Twenty proteins with the most relative importance in the prediction model were shown in Supplemental Fig. 3, of which, NEFL and GFAP had the highest importance.

### Associations of brain age gap with risk of dementia

**Fig. 1:**
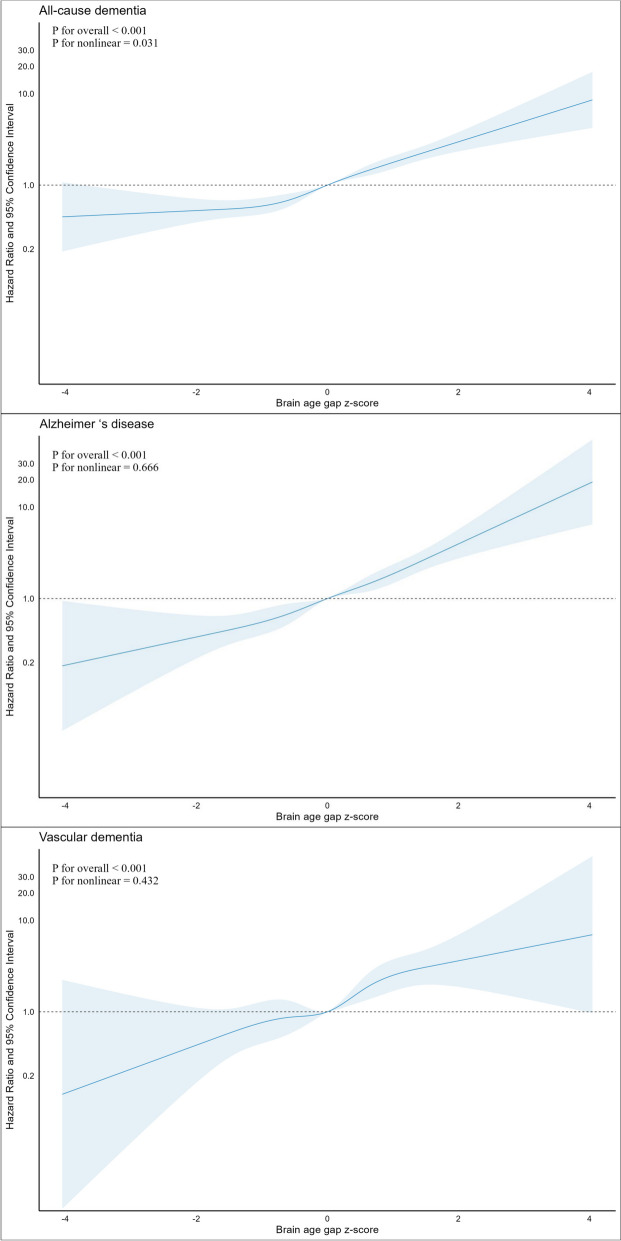
The adjusted hazard ratio of dementia by brain age gap as restricted cubic spline terms. The model adjusted for chronological age, sex, race, education levels, Townsend deprivation index, body mass index, smoking status, alcohol consumption, physical activity, healthy sleep patterns, healthy diet, social isolation, depression, hearing problems, hypertension, type 2 diabetes, high cholesterol, traumatic brain injury, history of CVD, NO_2_, PM_2.5_, and the number of *APOE* ε4 alleles

During a median follow-up of 13.75 years, a total of 1289 all-cause dementia, 575 AD, and 216 vascular diseases were identified. The results of the Schoenfeld residuals test did not reveal significant violations of the proportional hazards assumption by brain age gap with random scatter around zero and the lack of a significant trend in the smooth line (Supplemental Fig. 4). Table 2 shows the adjusted hazard ratios (HR) and 95% confidence intervals (CI) of dementia, according to per unit increment of brain age gap z-score and groups of extreme agers. We observed that the brain age gap was significantly associated with higher risks of all-cause dementia, AD, and vascular dementia in Model 2 (all *P*-values < 0.001). Further adjusting the number of *APOE* ε4 alleles resulted in the most conservative HRs (Model 3), that per-unit increment of brain age gap z-score was associated with 67% (HR [95%CI], 1.67 [1.56–1.79]; *P* < 0.001), 85% (1.85 [1.66–2.08]; *P* < 0.001), and 86% (1.86 [1.55–2.24]; *P* < 0.001) higher risks of all-cause dementia, AD, and vascular dementia, respectively. We observed a linear relationship between the brain age gap and risk of Alzheimer’s disease and vascular dementia (*P* for nonlinearity = 0.666 and 0.432, respectively, Fig. 1), with a slight flat trend for all-cause dementia (*P* for nonlinearity = 0.031) when brain age gap *z*-score <  − 2. Compared with non-extreme agers, extremely old agers were significantly associated with > threefold higher risks of all-cause dementia and vascular dementia (HR [95%CI], 3.42 [2.25–5.20], *P* < 0.001 and 3.41 [1.05–11.13], *P* = 0.042) and fourfold higher risk of AD (4.45 [2.32–8.54], *P* < 0.001). Similarly, extremely young agers had significantly lower risks of all-cause dementia and AD (HR [95%CI], 0.42 [0.25–0.73], *P* = 0.002 and 0.22 [0.07–0.70], *P* = 0.010), while lower but non-significant risk of vascular dementia (0.20 [0.03–1.45], *P* = 0.112) than non-extreme agers.

**Table 2:** The adjusted hazard ratio of dementia by brain age gap z-score and groups of extreme agers^a^

Outcomes	Per unit increment of *z*-score		Extreme agers				
			**Extreme young**		**Non-extreme**	**Extreme old**	
	**HR (95% CI)**	***P*-value**	**HR (95% CI)**	***P*-value**		**HR (95% CI)**	***P*-value**
**All-cause dementia**							
Model 1^b^	**1.78 (1.66–1.91)**	** < 0.001**	**0.37 (0.22–0.65)**	** < 0.001**	Ref	**4.44 (2.92–6.74)**	** < 0.001**
Model 2^c^	**1.70 (1.58–1.83)**	** < 0.001**	**0.41 (0.24–0.70)**	**0.001**	Ref	**3.75 (2.47–5.72)**	** < 0.001**
Model 3^d^	**1.67 (1.56–1.79)**	** < 0.001**	**0.42 (0.25–0.73)**	**0.002**	Ref	**3.42 (2.25–5.20)**	** < 0.001**
Model 4^e^	**1.69 (1.57–1.81)**	** < 0.001**	**0.41 (0.24–0.71)**	**0.001**	Ref	**3.72 (2.44–5.65)**	** < 0.001**
**Alzheimer’s disease**							
Model 1	**1.96 (1.76–2.20)**	** < 0.001**	**0.20 (0.07–0.63)**	**0.006**	Ref	**5.79 (3.02–11.09)**	** < 0.001**
Model 2	**1.91 (1.71–2.14)**	** < 0.001**	**0.22 (0.07–0.67)**	**0.008**	Ref	**5.23 (2.72–10.05)**	** < 0.001**
Model 3	**1.85 (1.66–2.08)**	** < 0.001**	**0.22 (0.07–0.70)**	**0.010**	Ref	**4.45 (2.32–8.54)**	** < 0.001**
Model 4	**1.88 (1.68–2.11)**	** < 0.001**	**0.21 (0.07–0.66)**	**0.008**	Ref	**5.09 (2.65–9.77)**	** < 0.001**
**Vascular dementia**							
Model 1	**2.05 (1.71–2.46)**	** < 0.001**	0.17 (0.02–1.21)	0.077	Ref	**4.64 (1.43–15.05)**	**0.011**
Model 2	**1.91 (1.59–2.30)**	** < 0.001**	0.19 (0.03–1.37)	0.100	Ref	**3.75 (1.15–12.24)**	**0.028**
Model 3	**1.86 (1.55–2.24)**	** < 0.001**	0.20 (0.03–1.45)	0.112	Ref	**3.41 (1.05–11.13)**	**0.042**
Model 4	**1.87 (1.56–2.25)**	** < 0.001**	0.19 (0.03–1.39)	0.102	Ref	**3.60 (1.11–11.72)**	**0.034**

CI, confidence interval; CVD, cardiovascular disease; HR, hazard ratio; NO_2_, nitrogen dioxide; PM_2.5_, particulate matter with diameters of ≤ 2.5 µm; PRS, polygenic risk score

^a^Extreme agers were defined as brain age gap *z*-score <  − 2 or > 2

^b^Model 1: chronological age and sex

^c^Model 2: model 1 + race, education levels, Townsend deprivation index, body mass index, smoking status, moderate alcohol consumption, physical activity, healthy sleep patterns, healthy diet, social isolation, depression, hearing problems, hypertension, type 2 diabetes, high cholesterol, traumatic brain injury, CVD history, NO_2_, and PM_2.5_

^d^Model 3: model 2 + the number of *APOE* ε4 alleles

^e^Model 4: model 2 + PRS for Alzheimer’s disease

Sensitivity analyses confirmed the robustness of the findings. The broader variability (SD of 3.85 years for the 127-protein brain age gap vs. 3.26 years for the 63-protein brain age gap) may have led to more pronounced associations with dementia risk, despite both models being comparable in overall predictive accuracy (Supplemental Table 6). Controlling the competing risk of all-cause mortality led to minimal attenuation of associations which indicated robustness (Supplemental Table 7). Among participants without any missing covariates (*n* = 25,154 for Model 3), we observed slight changes in HRs with a per-unit increment of brain age gap *z*-score (Supplemental Table 8). Similarly, adjusting for baseline vision problems did not significantly affect the associations of per-unit increment of brain age gap *z*-score (Supplemental Table 9). Although there were apparent changes in HRs with extreme agers in Supplement Tables 8 and 9, the main underlying reason should be a greatly reduced number of participants within extreme groups. Among participants ≥ 60 years old (Supplemental Table 10), the associations were consistent although slightly attenuated to less than 5% (e.g., HR for the per-unit increment of brain age gap *z*-score in Model 3, 1.67 [1.56–1.79] vs 1.62 [1.49–1.75]). Excluding the first 5-year follow-up resulted in apparent changes in HRs of extreme ages, indicating reverse causality, especially among extremely old participants (Supplemental Table 11). However, the consistently significant associations supported the robustness of the linkage between the brain age gap and dementia risk.

**Fig. 2:**
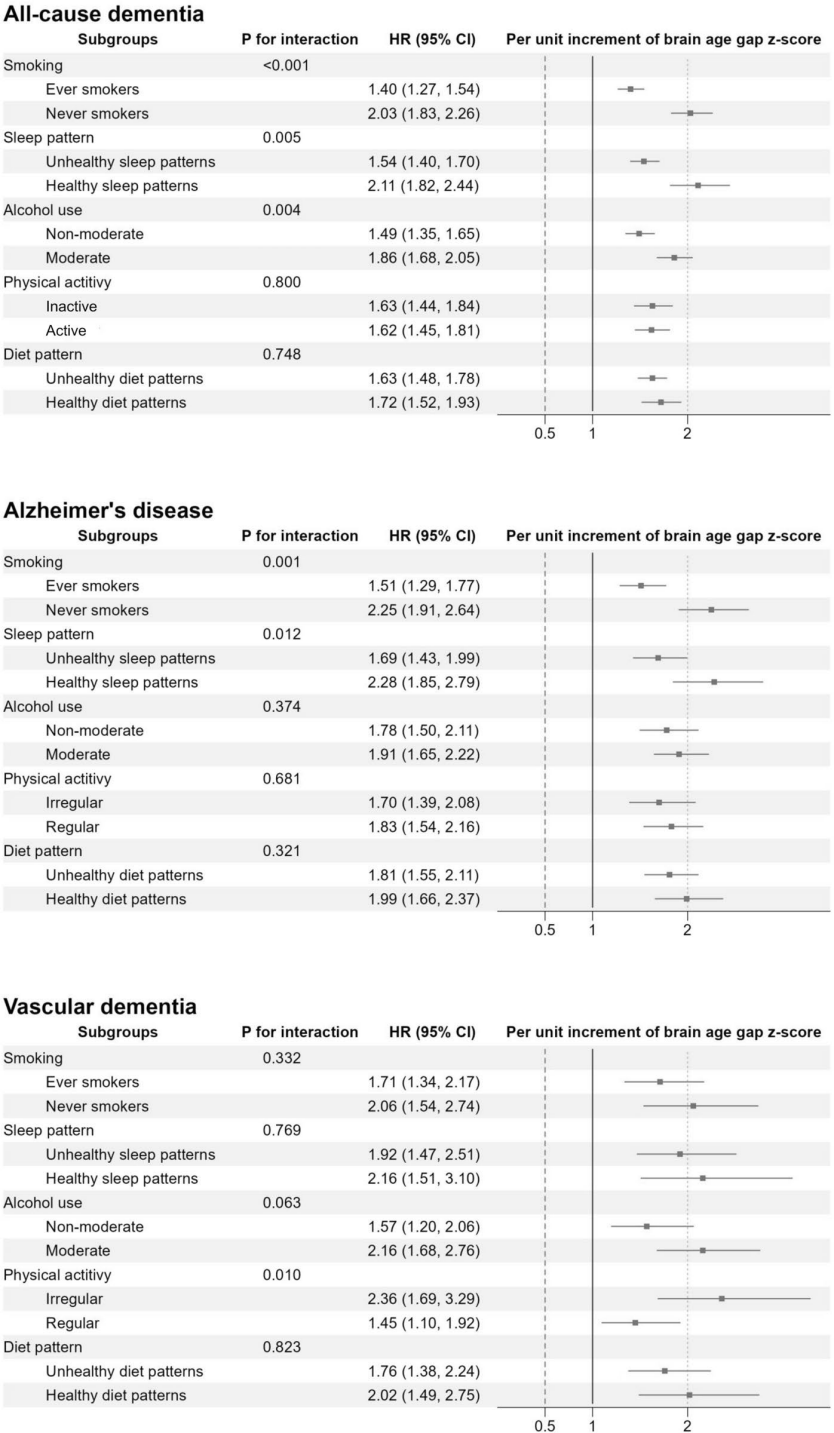
The adjusted hazard ratio of dementia for the per-unit increment of brain age gap *z*-score by subgroups of lifestyles factors. The model adjusted for chronological age, sex, race, education levels, Townsend deprivation index, body mass index, smoking status, alcohol consumption, physical activity, healthy sleep patterns, healthy diet, social isolation, depression, hearing problems, hypertension, type 2 diabetes, high cholesterol, traumatic brain injury, history of CVD, NO_2_, PM_2.5_, and the number of *APOE* ε4 alleles

We observed statistically significant interactions between the brain age gap and some lifestyle factors in relation to the risk of dementia (Fig. 2, *P* for interaction < 0.05). The associations between brain age gap and all-cause dementia were stronger among never smokers, moderate alcohol users, and those who had healthier sleep patterns (Fig. 2A). The association with AD was stronger among the never smokers and those who had healthier sleep patterns (Fig. 2B). As for vascular dementia, the association with brain gap was more pronounced among participants with inactive physical activity (Fig. 2C).

We also assessed the interaction of the brain age gap with genetic susceptibility to dementia (Supplemental Fig. 5) and observed significant interaction. There was a trend that the HR of all-cause dementia increased with the number of *APOE* ε4 alleles (*P* for interaction = 0.018).

### Associations of brain age gap with neuroimaging measurements

With a median interval of 9.10 years between baseline assessment and first imaging assessment, the associations between brain age gap and neuroimaging measurements are shown in Table 3. Per-unit increment of brain age gap z-score at baseline was related to lower volume of total brain, gray matter, peripheral cortical gray matter, and left hippocampus (Beta [SE], − 0.028 [0.014], *P* = 0.047, − 0.090 [0.013], *P* < 0.001, − 0.102 [0.013], *P* < 0.001, − 0.032 [0.016], *P* = 0.048), and higher volume of white matter, white matter hyperintensity, and ventricular cerebrospinal fluid (0.049 [0.016], *P* = 0.002, 0.084 [0.015], *P* < 0.001, and 0.067 [0.015], *P* < 0.001).

**Table 3:** The associations between brain age gap *z*-score and groups of extreme agers^a^ and neuroimaging measurements^b,c^

Neuroimaging measurements	Per unit increment of *z*-score		Extreme agers (*n* = 3695)				
			**Extreme young (*n* = 112)**		**Non-extreme**	**Extreme old (*n* = 72)**	
	**Beta (SE)**	***P*-values**	**Beta (SE)**	***P*-values**		**Beta (SE)**	***P*-values**
Volume of total brain	** − 0.028 (0.014)**	**0.047**	0.083 (0.082)	0.310	Ref	− 0.148 (0.103)	0.150
Volume of white matter	**0.049 (0.016)**	**0.002**	− 0.040 (0.095)	0.673	Ref	0.029 (0.119)	0.806
Volume of gray matter	** − 0.090 (0.013)**	** < 0.001**	**0.171 (0.074)**	**0.021**	Ref	** − 0.266 (0.093)**	**0.004**
Volume of peripheral cortical gray matter	** − 0.102 (0.013)**	** < 0.001**	**0.221 (0.077)**	**0.004**	Ref	** − 0.290 (0.096)**	**0.003**
Volume of white matter hyperintensity	**0.084 (0.015)**	** < 0.001**	** − 0.182 (0.086)**	**0.035**	Ref	**0.322 (0.110)**	**0.003**
Volume of ventricular cerebrospinal fluid	**0.067 (0.015)**	** < 0.001**	− 0.060 (0.089)	0.500	Ref	**0.284 (0.112)**	**0.011**
Volume of the left hippocampus	** − 0.032 (0.016)**	**0.048**	0.079 (0.094)	0.400	Ref	− 0.079 (0.118)	0.501
Volume of the right hippocampus	− 0.026 (0.016)	0.110	− 0.001 (0.095)	0.989	Ref	− 0.147 (0.119)	0.216
Volume of gray matter in the left hippocampus	− 0.025 (0.016)	0.108	− 0.043 (0.091)	0.640	Ref	− 0.120 (0.114)	0.296
Volume of gray matter in the right hippocampus	− 0.019 (0.016)	0.220	− 0.025 (0.091)	0.781	Ref	− 0.028 (0.114)	0.805

*CVD* cardiovascular disease, *NO*_*2*_ nitrogen dioxide, *PM*_*2.5*_ particulate matter with diameters of ≤ 2.5 µm, *SE* standard error

^a^Extreme agers were defined as brain age gap *z*-score <  − 2 or > 2

^b^Neuroimaging measurements were standardized with a mean of 0 and SD of 1

^c^Model adjusted for chronological age, sex, race, education levels, Townsend deprivation index, body mass index, smoking status, moderate alcohol consumption, physical activity, healthy sleep patterns, healthy diet, social isolation, depression, hearing problems, hypertension, type 2 diabetes, high cholesterol, traumatic brain injury, CVD history, NO_2_, PM_2.5_, the number of APOE ε4 alleles, prevalent Parkinson’s disease and multiple sclerosis, and time interval between baseline and MRI imaging assessment

## Discussion

In this prospective cohort study, we observed that plasma brain-enriched proteins accurately estimated brain aging, and the gap between proteomic brain age and chronological age was strongly associated with a higher risk of dementia, independent of chronological age and genetic susceptibility. These findings were corroborated by the associations between brain age gap and MRI-based brain volume measurements that reflected various dementia-related pathologies, including global and cortical atrophy, as well as white matter lesions. Furthermore, our results indicated that the associations between the brain age gap and dementia risk were modified by lifestyle factors and carriage of *APOE* ε4 alleles.

Our study enhances the current understanding of brain-specific aging and employs one of the largest proteomics datasets to date. While previous studies have developed proteomic age clocks with strong predictive values, they have not fully captured the differential organ-specific aging rate patterns [25–29]. In our study, we applied an innovative approach by focusing on brain-enriched proteomics to estimate brain-specific aging, building on the framework introduced by Oh et al. [7], which offered a novel perspective to explore brain aging independently of whole-body aging processes. Our method, leveraging a gradient-boosting machine learning framework and large sample size, might better handle non-linear relationships and complex interactions between proteins regarding the brain aging process. The application of the Boruta feature selection algorithm further refined our predictive model, ensuring that it emphasized proteins with the most valuable information for brain age prediction [30]. The greater predictive accuracy and generalizability of our chosen LightGBM method have been previously demonstrated in the UK Biobank, where it outperformed models like elastic net and neural networks in generalizing to independent datasets [6]. Of note, NEFL and GFAP showed the highest feature importance in predicting brain age in our model, supported by Guo et al.’s [31] proteome-wide association study, which identified NEFL and GFAP as key predictors of incident all-cause dementia, AD, and vascular dementia. While GDF15 and LTBP2 were highlighted in the Guo et al.[31] study, they are not included in our model as they are not classified as brain-enriched proteins. Nevertheless, the consistency in findings between the two studies strengthens the validity of our proteomic brain age model. These replicated associations across studies enhance the potential of plasma proteins like NEFL and GFAP as reliable biomarkers for predicting brain aging and dementia risk.

The current study suggests a robust and independent association of the brain-specific age gap with the risk of incident dementia, especially for AD. Extremely accelerated brain age conferred a > 400% higher risk of AD, while a > 300% higher risk of all-cause dementia as well as vascular dementia. The pathology of AD is intertwined with that of all-cause dementia. For example, multiple proteinopathies coexisted in human brains with dementia [32], including the presence of amyloid-β plaques and tau neurofibrillary tangles which biologically defined AD [33]. The findings of additional risk of AD versus all-cause dementia with the same proteomic brain age gap implied the complexity of dementia-related proteomic effects, and the potential of brain-specific proteomics to elucidate distinct pathological mechanisms. For example, cerebrovascular atherosclerosis is a shared risk factor for AD and vascular dementia [3, 34], and more predictive of vascular dementia [35, 36], which could be explained by differential proteomic effects. Recently, common proteomic effects between cerebral atherosclerosis and AD were found among 23 proteins [37], merely two of which (annotated gene NEFL, and QDPR) were brain-enriched and included in our analyses. Therefore, the remaining brain-enriched proteins were likely to indicate a brain aging process related to AD more than other types of dementia.

The proteomic brain age gap was related to the global pathophysiology of dementia, beyond chronological aging. MRI, as a neuroimaging marker of neurodegeneration, was used to assess macroscopic brain atrophy and assist in determining the etiology of dementia [4, 13], while lack of diagnostic usefulness due to the heterogeneity of pathophysiology, and mixed mechanisms involved [13]. For example, white matter hyperintensity is usually viewed as a vascular lesion and contributes to dementia, while emerging evidence suggests its relationship with AD pathology [38]. The associations of brain age gap with global atrophy (indicated by volume of total brain), cortical atrophy (indicated by volumes of gray matter), and white matter lesions thus should not be interpreted as proteomic effects preceding specific subtypes of dementia, but a comprehensive pattern of neurodegeneration. Intriguingly, in a population with normal cognition, gray and white matter showed a consistent age-dependent pattern of volume loss, and ventricular cerebrospinal fluid had a volume increase trend [39]. The associations of the brain age gap with brain volumes were in the same direction after adjusting chronological age, suggesting that the brain age gap might capture distinct biological aging processes beyond chronological aging.

The associations between the brain age gap and risk of dementia tended to be stronger among individuals with healthier lifestyles, such as never smoking, and healthy sleep patterns. Such observations might be explained through several potential mechanisms. First, individuals with unhealthy lifestyles, such as smoking or unhealthy sleep patterns, may have a higher baseline risk for dementia, potentially diminishing the relative contribution of the brain age gap. However, as we did not find significant differences in the variability of brain age gap across lifestyle groups, the stronger associations observed in healthier individuals were less likely to be explained by reduced variability in brain aging markers. Second, lifestyles might modulate biological resilience to withstand the consequences of neurodegenerative and cerebrovascular diseases that primarily precede dementia. For instance, deep sleep was found to increase resilience against amyloid-β [40], which was regulated by the sleep–wake cycle with higher clearance in sleep [41]. In individuals with healthier lifestyles, the increased resilience might allow the brain age gap to manifest more clearly in addition to primary biological biomarkers, amyloid-β plaques, and tau neurofibrillary tangles. Also, the observation implied that individuals with healthier lifestyles, especially never smoking and healthy sleep, would benefit more from controlling the proteomic brain aging process to prevent dementia. Third, the heterogeneity in the proteome might modify the relationship between the brain age gap and dementia risk. For instance, in ever smokers, the brain-enriched proteome may be impacted by smoking-related changes, potentially obscuring proteomic signatures of aging. In contrast, in never smokers, the proteomic brain age gap may more directly reflect aging processes associated with dementia risk. Given the absence of significant differences in variability between lifestyle groups, proteome-lifestyle interactions, rather than variability in brain aging itself, might account for the observed difference in associations. Fourth, the differential patterns of lifestyle interactions for AD and vascular dementia might suggest various underlying biological mechanisms, which warrant further investigation. However, all the interpretations need extra caution because no single explanation can be applied to all lifestyle factors.

The association between the brain age gap and dementia risk did not vary substantially according to genetic susceptibility. Although the association appeared stronger in homozygotes *APOE* ε4 carriers than non-carriers, the interaction was not statistically significant for either Alzheimer’s disease or vascular dementia. It remains biologically plausible that genetic susceptibility could interact with the proteomic brain age gap to influence dementia risk, through pathways involving amyloid-β accumulation, tau pathology, immune response, and lipid metabolism [42, 43]. One potential explanation for the lack of a significant interaction is insufficient statistical power, given the low prevalence of *APOE* ε4 allele carriers (~ 3%) and low incidence of dementia in this younger and healthier population. Alternatively, the lack of a significant interaction may suggest that the brain age gap captures broader neurodegenerative processes that are not directly linked to *APOE* ε4-related pathways. The idea aligns with previous findings that lifestyle factors and psychological traits relate to dementia risk independently of genetic determinants [11, 44]. Additionally, although *APOE* ε4 is consistently associated with lifespan and longevity [45], it is not included in either brain-specific or whole-body estimation model of proteomic age [6], suggesting that genetic and proteomic markers of aging may capture different dimensions of aging processes.

The major strengths of this study lie in its novel approach to studying brain aging through brain-enriched proteomics, the use of powerful machine learning methods, and a large community-based population. These elements collectively provide robust insights and offer a novel perspective for understanding the biological processes underlying brain aging. The study’s limitations also warrant consideration. First, the UK Biobank’s inherent “healthy volunteer” selection bias cannot be fully mitigated, which could potentially limit the generalizability of our findings. However, a recent study has proven the generalizability of whole-body proteomic age signatures with age-related diseases in different populations from the UK, China, and Finland [6], which may highly support the generalizability of our brain-specific proteomic age signature. Second, the Olink assay generated a smaller number of proteins as compared to SomaScan (2924 versus 4979 proteins totally measured, 127 versus 202 brain-enriched measured [7]) which might restrict the power of brain age prediction. However, the comparison between Olink and SomaScan platforms showed that Olink was better at capturing protein-trait associations [46]. Third, the low incidence of dementia, possibly due to the younger and healthier study population, may lead to an underestimation of the true association between the brain age gap and dementia risk. However, the consistency of findings across sensitivity analyses suggested the robustness, and the younger population might imply a special advantage in detecting early onset dementia. Fourth, we cannot fully rule out residual confounding effects from unmeasured lifestyle factors, such as diet quality and physical activity timing, despite the extensive adjustment of covariates. Future studies incorporating more detailed assessments of lifestyle factors are needed to further clarify their roles in brain aging and dementia.

In conclusion, our results indicate that the plasma proteomic brain age gap is a powerful biomarker of brain aging, and indicative of dementia risk and global neurodegeneration.

## Supplementary Information

Below is the link to the electronic supplementary material.

Supplementary file1 (DOCX 1137 KB)

## Data Availability

The UK Biobank is an open-access database, available by application.
