# Supplementary material for: Plasma proteomics-based brain aging signature and incident dementia risk
Source: GeroScience. 2024 Nov 12;47(2):2335–49. doi: 10.1007/s11357-024-01407-6 (PMC11978599; doi:10.1007/s11357-024-01407-6)
Supplement: Supplementary file 1 — Supplementary file1 (DOCX 1137 KB) [file 11357_2024_1407_MOESM1_ESM.docx]

**Plasma proteomics-based brain aging signature and incident dementia risk**

Minghao Kou, MHS^a^, Hao Ma, PhD^a^, Xuan Wang, PhD^a^, Yoriko Heianza, PhD,^a^ Lu Qi, PhD^a,b^

a. Department of Epidemiology, School of Public Health and Tropical Medicine, Tulane University, New Orleans, LA;

b. Department of Nutrition, Harvard T.H. Chan School of Public Health, Boston, MA

**Supplemental Methods**

**Proteomics data**

From April 2021 to January 2022, the UK Biobank Pharma Proteomics Project (UKB-PPP) consortium applied the Olink Explore Proximity Extension Assay across 53,014 unique UK Biobank participants, generating 2,924 unique protein measures. For the study, we only included participants measured from batch 1 to 6, which consisted of totally randomized participants at baseline (N = 45,429) and dropped batch 0 (pilot batch) and batch 7 (mix of randomized subsamples and COVID samples). The proteomics data was finally outlined in the Normalized Protein eXpression (NPX), Olink’s arbitrary unit in log_2_ scale. Detailed information of sample selection, quality control, and normalization could be found elsewhere.^1^

**Identification of brain-enriched proteins**

We defined brain-enriched proteins by identifying brain-enriched annotated genes using the Gene Tissue Expression Atlas (GTEx) human tissue bulk RNA-seq database.^2^ First, we normalized gene expression counts data using the DESeq2 R package^3^ to make comparison between tissues. Second, we grouped tissues of the same organ together and used the maximum expression counts as the gene’s expression level in a specific organ. Third, we defined a gene as brain-enriched gene if its expression is at least four times higher in brain than any other organs, following the definition proposed by the Human Protein Atlas.^4^ We finally identified 127 (4.4% of 2,916) proteins whose annotated genes’ expression were brain-enriched with protein names mapped through UniProt (<https://www.uniprot.org/>) (Supplemental Table 1). The method has been used elsewhere to identify organ-enriched proteins for multiple organs.^5^

**Proteomic brain age calculation**

First, we randomly split 45,429 participants into 70% training (N = 32,385) and 30% testing (N = 13,044) sets, and then trained a LightGBM machine learning model starting with 127 brain-enriched proteins to predict chronological age. The model hyperparameters were tuned via 5-fold cross validation using the Optuna module in Python. With 300 trials, the parameters were optimized to maximize the average R^2^ of the model across all folds. Second, we applied Boruta feature selection to select brain-enriched proteins with higher importance via shap-hypertune module with the best set of hyperparameters obtained earlier, with 200 trials and a threshold of 100% to select real features. Boruta is a robust feature selection technique that identifies relevant features by comparing them to randomized counterparts, ensuring that only the most informative and non-redundant proteins are selected. This reduction was aimed at improving model interpretability and reducing potential overfitting without sacrificing predictive accuracy. Third, we repeat the procedure for tuning hyperparameters in a new model with selected 63 proteins to obtain a new set of best parameters. Fourth, the performance of both tuned LightGBM models before and after feature selection were tested in the test set. Across all the abovementioned steps, the maximum run was set at 5000 with 20 early stopping rounds, and R^2^ as the evaluation metric to identify the model that explained the largest variation of chronological age. We then calculate brain age in the entire study population using 5-fold cross-validation before and after feature selection, respectively. A separate LightGBM model was trained with the best hyperparameters, and predicted age was estimated for the test set within each fold. We combined predicted age values from all 5 folds to create a measure named brain age for the entire study population. In the LightGBM model with 127 brain-enriched proteins to predict chronological age, the predicted brain ages with the best set of hyperparameters showed a strong correlation of 0.78 with chronological age (Supplemental Table 2). After Boruta feature selection, a total of 63 proteins were selected to fit a new LightGBM model and predict brain age, resulting in an even stronger correlation with chronological ages (r = 0.86). SHAP (SHapley Additive exPlanations)^6^ values were used to identify the relative importance of proteins in the prediction model. Therefore, the predicted brain age after feature selection was used for downstream analyses. Brain age calculation was carried out using Python v.3.6.11.

**Assessment of covariates**

A touch-screen questionnaire was used to assess most of the covariates at baseline.^7^ Sex was classified as men or women. Self-reported race was classified as White or non-White. We categorized the years of education into low (≤10 years), medium education (11-18 years), and high education (≥19 years), based on the highest qualification achieved.^8^ Townsend deprivation index is a composite measure of deprivation based on unemployment, non-car ownership, non-home ownership, and household overcrowding; a negative value represents high socioeconomic status. Body mass index was calculated as weight in kilograms divided by height in meters squared (kg/m²). Self-reported smoking status was classified into two categories: never smokers, and ever smokers. Alcohol intake was grouped into two categories: moderate consumption (defined as 0 to 14 g/d for women and 0 to 28 g/d for men, with the maximum limit reflecting US dietary guidelines),^9,10^ and non-moderate consumption (Supplemental Table 3). Physical activity was defined per the guideline as active (>150 minutes of moderate intensity activity per week or >75 minutes of vigorous activity per week or an equivalent combination per week), or inactive.^11^ Healthy diet was based on consumption of at least 4 of 7 food groups (fruits, vegetables, fish, processed meat, unprocessed red meat, whole grains, and refined grains) following recommendations on dietary priorities for cardiometabolic health (Supplemental Table 2).^12^ Healthy sleep patterns were based on having at least 4 of 5 healthier sleep factors (chronotype, duration, insomnia, snoring, and excessive daytime sleepiness) following previous studies in the UK Biobank (Supplemental Table 2).^13^ A social isolation score was used to evaluate isolation status based on previous UK biobank studies and social isolation was defined as isolation score ≥2.^14^ Self-reported depression was assessed with question: “Over past two weeks, how often have you felt down, depressed or hopeless?” We defined absence of depression if participants indicated “not at all”, and assigned missing value to those prefer not to answer. Hearing problems were assessed with the question: “Do you have any difficulty with your hearing?” We defined having hearing problem if participants answered “yes” or “I am completely deaf”, and assigned those prefer not to answer as missing value. Baseline hypertension was defined as a systolic blood pressure ≥140 mmHg or a diastolic blood pressure ≥90 mm Hg, a self-reported diagnosis by physicians, or blood pressure medication use. Baseline type 2 diabetes was ascertained based on ICD-10 codes (E11), a self-reported diagnosis by physicians or insulin use. Baseline high cholesterol was ascertained based on a self-reported diagnosis by physicians or lipid-lowering drug use. Traumatic brain injuries were defined as ICD-10 code S06 (“Intracranial injury”) before baseline. The history of cardiovascular diseases was defined by ICD-10 codes of coronary heart disease (I20-I25), stroke (I60-I64), heart failure (I11.0, I13.0, I13.2, I50.X), and atrial fibrillation (I48) before baseline. UK Biobank used a Land Use Regression model developed by the European Study of Cohorts for Air Pollution Effects (ESCAPE) project to calculate the annual average concentration of air pollution including particulate matter with diameters of ≤2.5 µm (PM_2.5_) and nitrogen dioxide (NO_2_).^15,16^ The exposure data of PM_2.5_ was collected in 2010, whereas the averaged values of NO_2_ between 2005-2007 and 2010 were used in the analysis. Vision problems were assessed with the question: “Has a doctor told you that you have any of the following problems with your eyes?” We defined having vision problem if participants answered, “Diabetes related eye disease”, “Glaucoma”, “Injury or trauma resulting in loss of vision”, “Cataract”, “Macular degeneration”, and “Other serious eye condition”. Prevalent Parkinson's disease and multiple sclerosis were defined by self-reported questionnaire and ICD-10 codes (Parkinson's disease, G20, G21, G21.0, G21.1, G21.2, G21.3, G21.4, G21.8, G21.9, G22, G23.0, G23.1, G23.2, G23.3, G23.8, G23.9, G25.9, G26, G90.3; multiple sclerosis, G35) happened before baseline.

**Supplemental Tables**

| **Supplemental Table 1. 127 brain-enriched proteins** | | | |
| --- | --- | --- | --- |
| **Abbreviation** | **UKB-PPP ProteinID** | **Protein name** | **Passing Boruta selection** |
| ABCA2 | ABCA2:Q9BZC7:OID30146:v1 | ATP-binding cassette sub-family A member 2 | Yes |
| ADAM22 | ADAM22:Q9P0K1:OID21001:v1 | Disintegrin and metalloproteinase domain-containing protein 22 | Yes |
| ADCYAP1R1 | ADCYAP1R1:P41586:OID21188:v1 | Pituitary adenylate cyclase-activating polypeptide type I receptor | No |
| AMBN | AMBN:Q9NP70:OID20437:v1 | Ameloblastin | Yes |
| ANGPTL7 | ANGPTL7:O43827:OID21412:v1 | Angiopoietin-related protein 7 | Yes |
| APLP1 | APLP1:P51693:OID20164:v1 | Amyloid-like protein 1 | Yes |
| APOD | APOD:P05090:OID30746:v1 | Apolipoprotein D | Yes |
| ATP1B2 | ATP1B2:P14415:OID30118:v1 | Sodium/potassium-transporting ATPase subunit beta-2 | No |
| ATP6V1G2 | ATP6V1G2:O95670:OID30167:v1 | V-type proton ATPase subunit G 2 | No |
| BCAN | BCAN:Q96GW7:OID20998:v1 | Brevican core protein | Yes |
| C1QL2 | C1QL2:Q7Z5L3:OID30515:v1 | Complement C1q-like protein 2 | Yes |
| CA11 | CA11:O75493:OID21195:v1 | Carbonic anhydrase-related protein 11 | Yes |
| CA14 | CA14:Q9ULX7:OID21401:v1 | Carbonic anhydrase 14 | Yes |
| CALCB | CALCB:P10092:OID31100:v1 | Calcitonin gene-related peptide 2 | Yes |
| CBLN1 | CBLN1:P23435:OID30851:v1 | Cerebellin-1 | No |
| CDH22 | CDH22:Q9UJ99:OID31332:v1 | Cadherin-22 | No |
| CEND1 | CEND1:Q8N111:OID30821:v1 | Cell cycle exit and neuronal differentiation protein 1 | No |
| CIT | CIT:O14578:OID30970:v1 | Citron Rho-interacting kinase | No |
| CLEC2L | CLEC2L:P0C7M8:OID30884:v1 | C-type lectin domain family 2 member L | No |
| CLIP2 | CLIP2:Q9UDT6:OID20559:v1 | CAP-Gly domain-containing linker protein 2 | No |
| CNDP1 | CNDP1:Q96KN2:OID20347:v1 | Beta-Ala-His dipeptidase | Yes |
| CNP | CNP:P09543:OID30285:v1 | 2',3'-cyclic-nucleotide 3'-phosphodiesterase | Yes |
| CNTF | CNTF:P26441:OID30807:v1 | Ciliary neurotrophic factor | No |
| CNTN1 | CNTN1:Q12860:OID20307:v1 | Contactin-1 | Yes |
| CNTN2 | CNTN2:Q02246:OID21426:v1 | Contactin-2 | Yes |
| CNTNAP2 | CNTNAP2:Q9UHC6:OID20556:v1 | Contactin-associated protein-like 2 | Yes |
| CNTNAP4 | CNTNAP4:Q9C0A0:OID30911:v1 | Contactin-associated protein-like 4 | No |
| CPLX2 | CPLX2:Q6PUV4:OID31186:v1 | Complexin-2 | No |
| CRH | CRH:P06850:OID21257:v1 | Corticoliberin | No |
| CRTAM | CRTAM:O95727:OID20914:v1 | Cytotoxic and regulatory T-cell molecule | Yes |
| CSPG5 | CSPG5:O95196:OID30843:v1 | Chondroitin sulfate proteoglycan 5 | No |
| DNAJC6 | DNAJC6:O75061:OID30247:v1 | Putative tyrosine-protein phosphatase auxilin | No |
| DNM1 | DNM1:Q05193:OID31458:v1 | Dynamin-1 | No |
| DNM3 | DNM3:Q9UQ16:OID31000:v1 | Dynamin-3 | Yes |
| ELAVL4 | ELAVL4:P26378:OID30854:v1 | ELAV-like protein 4 | No |
| ENO2 | ENO2:P09104:OID21046:v1 | Gamma-enolase | No |
| ENOPH1 | ENOPH1:Q9UHY7:OID31349:v1 | Enolase-phosphatase E1 | No |
| ENPP6 | ENPP6:Q6UWR7:OID30273:v1 | Glycerophosphocholine cholinephosphodiesterase ENPP6 | Yes |
| EXTL1 | EXTL1:Q92935:OID30114:v1 | Exostosin-like 1 | No |
| FABP6 | FABP6:P51161:OID20076:v1 | Gastrotropin | Yes |
| FGF3 | FGF3:P11487:OID30544:v1 | Fibroblast growth factor 3 | No |
| FGFR2 | FGFR2:P21802:OID21478:v1 | Fibroblast growth factor receptor 2 | Yes |
| FKBP1B | FKBP1B:P68106:OID20618:v1 | Peptidyl-prolyl cis-trans isomerase FKBP1B | Yes |
| FLT3 | FLT3:P36888:OID21272:v1 | Receptor-type tyrosine-protein kinase FLT3 | Yes |
| GABRA4 | GABRA4:P48169:OID30803:v1 | Gamma-aminobutyric acid receptor subunit alpha-4 | Yes |
| GAD1 | GAD1:Q99259:OID31166:v1 | Glutamate decarboxylase 1 | No |
| GAD2 | GAD2:Q05329:OID30449:v1 | Glutamate decarboxylase 2 | Yes |
| GFAP | GFAP:P14136:OID21247:v1 | Glial fibrillary acidic protein | Yes |
| GPC5 | GPC5:P78333:OID20944:v1 | Glypican-5 | Yes |
| GPR101 | GPR101:Q96P66:OID30819:v1 | Probable G-protein coupled receptor 101 | Yes |
| GPR158 | GPR158:Q5T848:OID31013:v1 | Probable G-protein coupled receptor 158 | No |
| GPR37 | GPR37:O15354:OID20095:v1 | Prosaposin receptor GPR37 | Yes |
| GRIK2 | GRIK2:Q13002:OID30977:v1 | Glutamate receptor ionotropic, kainate 2 | Yes |
| GRIN2B | GRIN2B:Q13224:OID30802:v1 | Glutamate receptor ionotropic, NMDA 2B | No |
| HTR1A | HTR1A:P08908:OID30824:v1 | 5-hydroxytryptamine receptor 1A | No |
| IDS | IDS:P22304:OID20619:v1 | Iduronate 2-sulfatase | Yes |
| IGLON5 | IGLON5:A6NGN9:OID31169:v1 | IgLON family member 5 | No |
| IGSF21 | IGSF21:Q96ID5:OID30210:v1 | Immunoglobulin superfamily member 21 | Yes |
| IL9 | IL9:P15248:OID31298:v1 | Interleukin-9 | No |
| IMPG1 | IMPG1:Q17R60:OID30814:v1 | Interphotoreceptor matrix proteoglycan 1 | No |
| KCNIP4 | KCNIP4:Q6PIL6:OID20813:v1 | Kv channel-interacting protein 4 | Yes |
| KIAA1549L | KIAA1549L:Q6ZVL6:OID31312:v1 | UPF0606 protein KIAA1549L | Yes |
| KLK6 | KLK6:Q92876:OID21526:v1 | Kallikrein-6 | Yes |
| LCN15 | LCN15:Q6UWW0:OID31032:v1 | Lipocalin-15 | Yes |
| LHPP | LHPP:Q9H008:OID20749:v1 | Phospholysine phosphohistidine inorganic pyrophosphate phosphatase | Yes |
| LRFN2 | LRFN2:Q9ULH4:OID30895:v1 | Leucine-rich repeat and fibronectin type-III domain-containing protein 2 | Yes |
| LRTM2 | LRTM2:Q8N967:OID31020:v1 | Leucine-rich repeat and transmembrane domain-containing protein 2 | Yes |
| MAG | MAG:P20916:OID30967:v1 | Myelin-associated glycoprotein | No |
| MAP2 | MAP2:P11137:OID31138:v1 | Microtubule-associated protein 2 | No |
| MDGA1 | MDGA1:Q8NFP4:OID20951:v1 | MAM domain-containing glycosylphosphatidylinositol anchor protein 1 | Yes |
| MEGF10 | MEGF10:Q96KG7:OID20746:v1 | Multiple epidermal growth factor-like domains protein 10 | Yes |
| MEGF11 | MEGF11:A6BM72:OID30099:v1 | Multiple epidermal growth factor-like domains protein 11 | No |
| MEPE | MEPE:Q9NQ76:OID20753:v1 | Matrix extracellular phosphoglycoprotein | Yes |
| MOG | MOG:Q16653:OID21384:v1 | Myelin-oligodendrocyte glycoprotein | Yes |
| NCAN | NCAN:O14594:OID21055:v1 | Neurocan core protein | Yes |
| NEFL | NEFL:P07196:OID20871:v1 | Neurofilament light polypeptide | Yes |
| NPTX1 | NPTX1:Q15818:OID21074:v1 | Neuronal pentraxin-1 | Yes |
| NPTXR | NPTXR:O95502:OID20191:v1 | Neuronal pentraxin receptor | Yes |
| NRGN | NRGN:Q92686:OID30674:v1 | Neurogranin | No |
| NRXN3 | NRXN3:Q9Y4C0:OID30901:v1 | Neurexin-3 | No |
| NTRK2 | NTRK2:Q16620:OID20136:v1 | BDNF/NT-3 growth factors receptor | Yes |
| OMG | OMG:P23515:OID21352:v1 | Oligodendrocyte-myelin glycoprotein | Yes |
| OXT | OXT:P01178:OID21047:v1 | Oxytocin-neurophysin 1 | Yes |
| PENK | PENK:P01210:OID30666:v1 | Proenkephalin-A | Yes |
| PMCH | PMCH:P20382:OID30172:v1 | Pro-MCH | No |
| PNMA2 | PNMA2:Q9UL42:OID30842:v1 | Paraneoplastic antigen Ma2 | No |
| POLR2F | POLR2F:P61218:OID21283:v1 | DNA-directed RNA polymerases I, II, and III subunit RPABC2 | Yes |
| PPP3R1 | PPP3R1:P63098:OID20902:v1 | Calcineurin subunit B type 1 | Yes |
| PSRC1 | PSRC1:Q6PGN9:OID21169:v1 | Proline/serine-rich coiled-coil protein 1 | No |
| PTPRR | PTPRR:Q15256:OID31099:v1 | Receptor-type tyrosine-protein phosphatase R | Yes |
| PTPRZ1 | PTPRZ1:P23471:OID30343:v1 | Receptor-type tyrosine-protein phosphatase zeta | Yes |
| PVALB | PVALB:P20472:OID21420:v1 | Parvalbumin alpha | No |
| QDPR | QDPR:P09417:OID20174:v1 | Dihydropteridine reductase | No |
| RAB37 | RAB37:Q96AX2:OID20425:v1 | Ras-related protein Rab-37 | Yes |
| RAB6B | RAB6B:Q9NRW1:OID20817:v1 | Ras-related protein Rab-6B | No |
| RASGRF1 | RASGRF1:Q13972:OID31213:v1 | Ras-specific guanine nucleotide-releasing factor 1 | No |
| RASSF2 | RASSF2:P50749:OID21194:v1 | Ras association domain-containing protein 2 | No |
| RGS8 | RGS8:P57771:OID20454:v1 | Regulator of G-protein signaling 8 | No |
| RTN4R | RTN4R:Q9BZR6:OID21424:v1 | Reticulon-4 receptor | No |
| SCN2A | SCN2A:Q99250:OID30833:v1 | Sodium channel protein type 2 subunit alpha | No |
| SCN2B | SCN2B:O60939:OID30948:v1 | Sodium channel subunit beta-2 | No |
| SCN4B | SCN4B:Q8IWT1:OID30277:v1 | Sodium channel subunit beta-4 | No |
| SCRN1 | SCRN1:Q12765:OID20542:v1 | Secernin-1 | No |
| SEMA4D | SEMA4D:Q92854:OID21020:v1 | Semaphorin-4D | Yes |
| SERPINI1 | SERPINI1:Q99574:OID30604:v1 | Neuroserpin | No |
| SEZ6 | SEZ6:Q53EL9:OID30968:v1 | Seizure protein 6 homolog | No |
| SEZ6L | SEZ6L:Q9BYH1:OID21488:v1 | Seizure 6-like protein | No |
| SLITRK1 | SLITRK1:Q96PX8:OID30510:v1 | SLIT and NTRK-like protein 1 | Yes |
| SNAP25 | SNAP25:P60880:OID30811:v1 | Synaptosomal-associated protein 25 | Yes |
| SNCA | SNCA:P37840:OID30645:v1 | Alpha-synuclein | No |
| SOWAHA | SOWAHA:Q2M3V2:OID30888:v1 | Ankyrin repeat domain-containing protein SOWAHA | No |
| SPOCK1 | SPOCK1:Q08629:OID20924:v1 | Testican-1 | Yes |
| STX1B | STX1B:P61266:OID30934:v1 | Syntaxin-1B | No |
| SV2A | SV2A:Q7L0J3:OID30965:v1 | Synaptic vesicle glycoprotein 2A | No |
| SYT1 | SYT1:P21579:OID31089:v1 | Synaptotagmin-1 | No |
| TAGLN3 | TAGLN3:Q9UI15:OID31225:v1 | Transgelin-3 | No |
| TBR1 | TBR1:Q16650:OID30923:v1 | T-box brain protein 1 | No |
| THY1 | THY1:P04216:OID21050:v1 | Thy-1 membrane glycoprotein | Yes |
| TMPRSS5 | TMPRSS5:Q9H3S3:OID20947:v1 | Transmembrane protease serine 5 | No |
| TNR | TNR:Q92752:OID20957:v1 | Tenascin-R | No |
| TREM2 | TREM2:Q9NZC2:OID20731:v1 | Triggering receptor expressed on myeloid cells 2 | Yes |
| TSPAN7 | TSPAN7:P41732:OID30810:v1 | Tetraspanin-7 | No |
| TUBB3 | TUBB3:Q13509:OID30799:v1 | Tubulin beta-3 chain | No |
| VSNL1 | VSNL1:P62760:OID31105:v1 | Visinin-like protein 1 | Yes |
| VSTM2B | VSTM2B:A6NLU5:OID31272:v1 | V-set and transmembrane domain-containing protein 2B | No |
| VWC2L | VWC2L:B2RUY7:OID31002:v1 | von Willebrand factor C domain-containing protein 2-like | No |
| WASF3 | WASF3:Q9UPY6:OID20882:v1 | Wiskott-Aldrich syndrome protein family member 3 | No |
| Abbreviation: UKB-PPP, UK Biobank Pharma Proteomics Project | | | |

| **Supplemental Table 2. Pearson's correlation between brain age, brain age gap and chronological age** | | | | |
| --- | --- | --- | --- | --- |
|  | **Chronological age** | **Brain age  based on 127 proteins** | **Brain age  based on 63 proteins** | **Brain age gap** |
| **Chronological age** | 1 |  |  |  |
| **Brain age based on 127 proteins** | 0.78 | 1 |  |  |
|  | P < 0.0001 |  |  |  |
| **Brain age  based on 63 proteins** | 0.86 | 0.97 | 1 |  |
|  | P < 0.0001 | P < 0.0001 |  |  |
| **Brain age gap** | <0.001 | 0.59 | 0.51 | 1 |
|  | P = 0.87 | P < 0.0001 | P < 0.0001 |  |

| **Supplemental Table 3. Definition of lifestyle factors** | | |
| --- | --- | --- |
| **Lifestyle factors** | **Categories** | **Values assigned** |
| Smoking status |  |  |
|  | - Never smoker | 1 |
|  | - Previous smoker | 0 |
|  | - Current smoker | 0 |
| Alcohol consumption | Drink-equivalents were calculated per guidelines, by multiplying the volume in ounces by the alcohol content in percent and dividing by 0.6 ounces of alcohol per drink-equivalent: 125ml wine=0.85 drink-equivalents, 4% ABV pint beer = 1.28 drink-equivalents, 25ml spirits=0.57 drink-equivalents, 50ml fortified wine= 0.56 drink-equivalents. Then drink-equivalents were converted to grams:  1 drink-equivalent = 14g of pure alcohol. |  |
|  | - For men, 0 to 28 g/d | 1 |
|  | - For women, 0 to 14 g/d | 1 |
|  | - Others | 0 |
| Regular physical activity |  |  |
|  | - ≥150 minutes of moderate intensity activity or ≥75 minutes of vigorous activity or an equivalent combination per week | 1 |
|  | - <150 minutes of moderate intensity activity and <75 minutes of vigorous activity or an equivalent combination per week | 0 |
| Healthy diet | Healthy diet was based on consumption of at least 4 of 7 food groups |  |
|  | - Fruits: ≥ 3 servings/day | 1 |
|  | - Vegetables: ≥ 3 servings/day | 1 |
|  | - Fish: ≥2 servings/week | 1 |
|  | - Processed meats: ≤ 1 serving/week | 1 |
|  | - Unprocessed red meats: ≤ 1.5 servings/week | 1 |
|  | - Whole grains: ≥ 3servings/day | 1 |
|  | - Refined grains: ≤1.5servings/day | 1 |
|  | - Others | 0 |
| Healthy sleep pattern | Healthy sleep pattern was based on at least 4 of 5 healthier sleep factors |  |
|  | - Chronotype: morning, or morning than evening | 1 |
|  | - Sleep duration: 7-8 h per day | 1 |
|  | - Insomnia: never, or rarely insomnia symptoms | 1 |
|  | - Snoring: no self-reported snoring | 1 |
|  | - Day-time sleepiness: never, rarely, or sometimes | 1 |
|  | - Others | 0 |

| **Supplemental Table 4. Baseline characteristics of the study participants by brain age gap** | | | | | |
| --- | --- | --- | --- | --- | --- |
| **Baseline characteristics** | **Total  (n = 45374)** | **Extreme young  (n=1189)** | **Non-extreme  (n=43208)** | **Extreme old  (n=977)** | **P-value^a^** |
| Chronological age, years | 56.8 (8.19) | 57.3 (7.01) | 57.0 (8.19) | 49.5 (5.69) | < 0.001 |
| Men, % | 45.9 | 50.5 | 45.8 | 44.2 | 0.003 |
| White, % | 93.4 | 92.7 | 93.6 | 87.5 | < 0.001 |
| Body mass index, kg/m² | 27.5 (4.81) | 27.1 (4.34) | 27.5 (4.81) | 27.9 (5.43) | 0.001 |
| Education level |  |  |  |  | 0.002 |
| - Low, % | 34.8 | 30.4 | 35.0 | 33.2 |  |
| - Medium, % | 17.5 | 16.9 | 17.6 | 16.1 |  |
| - High, % | 47.6 | 52.7 | 47.4 | 50.7 |  |
| Townsend deprivation index | -1.2 (3.20) | -1.5 (3.06) | -1.2 (3.19) | 0.0 (3.57) | < 0.001 |
| Never smoking, % | 54.1 | 58.9 | 54.1 | 51.2 | 0.001 |
| Moderate alcohol consumption, % | 52.7 | 53.1 | 52.7 | 52.8 | 0.956 |
| Physical active, % | 58.6 | 61.5 | 58.6 | 52.8 | 0.001 |
| Healthy diet, % | 36.7 | 35.2 | 36.8 | 32.1 | 0.008 |
| Healthy sleep pattern, % | 37.0 | 40.4 | 37.0 | 30.8 | < 0.001 |
| Hypertension, % | 56.0 | 51.2 | 56.3 | 48.0 | < 0.001 |
| Type 2 diabetes, % | 3.2 | 1.4 | 3.2 | 5.8 | < 0.001 |
| High cholesterol, % | 19.8 | 15.7 | 20.0 | 15.4 | < 0.001 |
| Social isolation, % | 14.3 | 11.8 | 14.3 | 19.6 | < 0.001 |
| Depression, % | 24.3 | 22.3 | 24.1 | 38.0 | < 0.001 |
| Hearing problems, % | 25.7 | 25.2 | 25.8 | 21.4 | 0.010 |
| CVD history, % | 8.9 | 5.9 | 9.0 | 8.3 | 0.001 |
| Traumatic brain injury, % | 0.1 | 0.1 | 0.1 | 0.2 | 0.157 |
| NO_2_, µg/m^3^ | 29.4 (9.33) | 29.0 (9.88) | 29.4 (9.31) | 30.8 (9.49) | < 0.001 |
| PM_2.5_, µg/m^3^ | 10.0 (1.07) | 10.0 (1.09) | 10.0 (1.07) | 10.2 (1.09) | < 0.001 |
| Number of the APOE e4 alleles |  |  |  |  | 0.295 |
| - 0, % | 70.8 | 69.4 | 70.8 | 73.0 |  |
| - 1, % | 26.2 | 28.0 | 26.2 | 24.5 |  |
| - 2, % | 3.0 | 2.5 | 3.0 | 2.5 |  |
| PRS for Alzheimer’s disease |  |  |  |  | 0.603 |
| - Low, % | 33.0 | 32.7 | 33.0 | 34.4 |  |
| - Medium, % | 33.2 | 33.2 | 33.2 | 34.3 |  |
| - High, % | 33.7 | 34.1 | 33.8 | 31.3 |  |
| Abbreviation: CVD, cardiovascular disease; NO2, nitrogen dioxide; PM2.5, particulate matter with diameters of ≤2.5 µm; PRS, polygenic risk score | | | | | |
| a. P-values were tested by ANOVA F-test for continuous variables, and Chi-Square test for categorical variables | | | | | |

| **Supplemental Table 5. Model performance for estimation of proteomic brain age in subgroups of baseline characteristics** | | | |
| --- | --- | --- | --- |
| **Baseline characteristics** | **Pearson's correlation** | **R^2^** | **Root mean square error** |
| Sex |  |  |  |
| Women | 0.87 | 0.75 | 4.05 |
| Men | 0.85 | 0.73 | 4.36 |
| Race/ethnicity |  |  |  |
| White | 0.86 | 0.74 | 4.16 |
| Mixed | 0.83 | 0.69 | 4.49 |
| Asian | 0.85 | 0.72 | 4.47 |
| Black | 0.82 | 0.67 | 4.56 |
| Others | 0.82 | 0.67 | 4.62 |
| Body mass index |  |  |  |
| 0-18.5 | 0.84 | 0.70 | 4.39 |
| 18.5-25 | 0.87 | 0.75 | 4.14 |
| 25-30 | 0.86 | 0.73 | 4.21 |
| ≥30 | 0.85 | 0.72 | 4.24 |
| Education level |  |  |  |
| Low | 0.85 | 0.72 | 4.16 |
| Medium | 0.86 | 0.74 | 4.10 |
| High | 0.85 | 0.73 | 4.25 |
| Townsend deprivation index |  |  |  |
| ≤Median | 0.86 | 0.74 | 4.10 |
| >Median | 0.86 | 0.74 | 4.27 |
| Never smoking |  |  |  |
| No | 0.85 | 0.72 | 4.24 |
| Yes | 0.86 | 0.75 | 4.17 |
| Moderate alcohol consumption |  |  |  |
| No | 0.85 | 0.73 | 4.22 |
| Yes | 0.86 | 0.74 | 4.18 |
| Physical active |  |  |  |
| No | 0.85 | 0.73 | 4.19 |
| Yes | 0.86 | 0.75 | 4.19 |
| Healthy diet |  |  |  |
| No | 0.86 | 0.74 | 4.23 |
| Yes | 0.86 | 0.73 | 4.10 |
| Healthy sleep pattern |  |  |  |
| No | 0.85 | 0.73 | 4.21 |
| Yes | 0.87 | 0.76 | 4.11 |
| Social isolation |  |  |  |
| No | 0.86 | 0.74 | 4.19 |
| Yes | 0.86 | 0.73 | 4.18 |
| Depression |  |  |  |
| No | 0.86 | 0.74 | 4.13 |
| Yes | 0.85 | 0.72 | 4.32 |
| Hypertension |  |  |  |
| No | 0.86 | 0.73 | 4.21 |
| Yes | 0.83 | 0.70 | 4.13 |
| Type 2 diabetes |  |  |  |
| No | 0.86 | 0.74 | 4.19 |
| Yes | 0.80 | 0.64 | 4.37 |
| High cholesterol |  |  |  |
| No | 0.86 | 0.74 | 4.21 |
| Yes | 0.78 | 0.61 | 4.04 |
| Hearing problems |  |  |  |
| No | 0.86 | 0.74 | 4.20 |
| Yes | 0.83 | 0.69 | 4.13 |
| CVD history |  |  |  |
| No | 0.86 | 0.74 | 4.21 |
| Yes | 0.77 | 0.60 | 4.05 |
| Number of the APOE e4 alleles |  |  |  |
| 0 | 0.86 | 0.73 | 4.23 |
| 1 | 0.86 | 0.75 | 4.10 |
| 2 | 0.88 | 0.78 | 4.17 |
| PRS for Alzheimer’s disease |  |  |  |
| Low | 0.85 | 0.73 | 4.26 |
| Medium | 0.86 | 0.74 | 4.18 |
| High | 0.86 | 0.74 | 4.16 |
| Abbreviation: CVD, cardiovascular disease; PRS, polygenic risk score | | | |

| **Supplemental Table 6. The adjusted hazard ratio of dementia by brain age gap z-score and groups of extreme agers^a^ estimated by all 127 brain-enriched proteins** | | | | | | | |
| --- | --- | --- | --- | --- | --- | --- | --- |
| **Outcomes** | **Per unit increment of z-score** | | **Extreme agers** | | | | |
|  |  |  | **Extreme young** | | **Non-extreme** | **Extreme old** | |
|  | **HR (95% CI)** | **P-value** | **HR (95% CI)** | **P-value** |  | **HR (95% CI)** | **P-value** |
| **All-cause dementia** |  |  |  |  |  |  |  |
| Model 1^b^ | **1.96 (1.82-2.10)** | **<0.001** | **0.33 (0.18-0.59)** | **<0.001** | ref | **5.66 (3.75-8.53)** | **<0.001** |
| Model 2^c^ | **1.86 (1.73-2.00)** | **<0.001** | **0.36 (0.20-0.66)** | **0.001** | ref | **4.52 (2.99-6.84)** | **<0.001** |
| Model 3^d^ | **1.81 (1.68-1.94)** | **<0.001** | **0.38 (0.21-0.68)** | **0.001** | ref | **4.11 (2.71-6.21)** | **<0.001** |
| Model 4^e^ | **1.83 (1.71-1.97)** | **<0.001** | **0.37 (0.20-0.66)** | **0.001** | ref | **4.37 (2.89-6.62)** | **<0.001** |
| **Alzheimer’s disease** |  |  |  |  |  |  |  |
| Model 1 | **2.19 (1.95-2.45)** | **<0.001** | **0.14 (0.04-0.58)** | **0.006** | ref | **6.41 (3.24-12.69)** | **<0.001** |
| Model 2 | **2.13 (1.90-2.39)** | **<0.001** | **0.16 (0.04-0.63)** | **0.009** | ref | **5.46 (2.75-10.85)** | **<0.001** |
| Model 3 | **2.01 (1.79-2.25)** | **<0.001** | **0.17 (0.04-0.67)** | **0.011** | ref | **4.63 (2.33-9.19)** | **<0.001** |
| Model 4 | **2.06 (1.84-2.31)** | **<0.001** | **0.16 (0.04-0.64)** | **0.009** | ref | **5.08 (2.56-10.08)** | **<0.001** |
| **Vascular dementia** |  |  |  |  |  |  |  |
| Model 1 | **2.29 (1.91-2.76)** | **<0.001** | 0.17 (0.02-1.24) | 0.080 | ref | **3.62 (0.87-15.02)** | **0.076** |
| Model 2 | **2.14 (1.78-2.57)** | **<0.001** | 0.20 (0.03-1.45) | 0.112 | ref | **2.80 (0.67-11.69)** | **0.158** |
| Model 3 | **2.05 (1.71-2.47)** | **<0.001** | 0.21 (0.03-1.52) | 0.123 | ref | **2.54 (0.61-10.63)** | **0.202** |
| Model 4 | **2.06 (1.72-2.48)** | **<0.001** | 0.21 (0.03-1.48) | 0.117 | ref | **2.60 (0.62-10.85)** | **0.191** |
| Abbreviation: CI, confidence interval; CVD, cardiovascular disease; HR, hazard ratio; NO_2_, nitrogen dioxide; PM_2.5_, particulate matter with diameters of ≤2.5 µm; PRS, polygenic risk score | | | | | | | |
| a. Extreme agers were defined as brain age gap z-score <-2 or > 2 | | | | | | | |
| b. Model 1: chronological age and sex | | | | | | | |
| c. Model 2: model 1 + race, education levels, Townsend deprivation index, body mass index, smoking status, moderate alcohol consumption, regular physical activity, healthy sleep patterns, healthy diet, social isolation, depression, hearing problems, hypertension, type 2 diabetes, high cholesterol, traumatic brain injury, CVD history, NO_2_, and PM_2.5_ | | | | | | | |
| d. Model 3: model 2 + the number of *APOE* ε4 alleles | | | | | | | |
| e. Model 4: model 2 + PRS for Alzheimer's disease | | | | | | | |

| **Supplemental Table 7. The adjusted hazard ratio of dementia by brain age gap z-score and extreme agers^a^ after accounting for competing risk of all-cause mortality** | | | | | | | |
| --- | --- | --- | --- | --- | --- | --- | --- |
| **Outcomes** | **Per unit increment of z-score** | | **Extreme agers** | | | | |
|  |  |  | **Extreme young** | | **Non-extreme** | **Extreme old** | |
|  | **HR (95% CI)** | **P-value** | **HR (95% CI)** | **P-value** |  | **HR (95% CI)** | **P-value** |
| **All-cause dementia** |  |  |  |  |  |  |  |
| Model 1^b^ | **1.72 (1.60-1.85)** | **<0.001** | **0.40 (0.23-0.68)** | **0.001** | ref | **4.19 (2.74-6.39)** | **<0.001** |
| Model 2^c^ | **1.65 (1.53-1.78)** | **<0.001** | **0.43 (0.25-0.74)** | **0.002** | ref | **3.65 (2.37-5.60)** | **<0.001** |
| Model 3^d^ | **1.63 (1.52-1.76)** | **<0.001** | **0.44 (0.26-0.77)** | **0.004** | ref | **3.38 (2.18-5.22)** | **<0.001** |
| Model 4^e^ | **1.64 (1.52-1.77)** | **<0.001** | **0.43 (0.25-0.74)** | **0.003** | ref | **3.59 (2.34-5.52)** | **<0.001** |
| **Alzheimer’s disease** |  |  |  |  |  |  |  |
| Model 1 | **1.88 (1.68-2.11)** | **<0.001** | **0.22 (0.07-0.67)** | **0.008** | ref | **5.42 (2.83-10.37)** | **<0.001** |
| Model 2 | **1.85 (1.65-2.08)** | **<0.001** | **0.23 (0.07-0.70)** | **0.010** | ref | **5.05 (2.61-9.77)** | **<0.001** |
| Model 3 | **1.80 (1.60-2.02)** | **<0.001** | **0.23 (0.07-0.74)** | **0.013** | ref | **4.41 (2.27-8.55)** | **<0.001** |
| Model 4 | **1.82 (1.62-2.04)** | **<0.001** | **0.23 (0.07-0.71)** | **0.010** | ref | **4.91 (2.53-9.52)** | **<0.001** |
| **Vascular dementia** |  |  |  |  |  |  |  |
| Model 1 | **1.95 (1.61-2.36)** | **<0.001** | 0.18 (0.03-1.30) | 0.089 | ref | **4.30 (1.30-14.18)** | **0.017** |
| Model 2 | **1.83 (1.51-2.22)** | **<0.001** | 0.21 (0.03-1.46) | 0.114 | ref | **3.54 (1.06-11.84)** | **0.040** |
| Model 3 | **1.79 (1.48-2.17)** | **<0.001** | 0.22 (0.03-1.55) | 0.128 | ref | **3.30 (0.98-11.06)** | **0.053** |
| Model 4 | **1.80 (1.49-2.17)** | **<0.001** | 0.21 (0.03-1.49) | 0.119 | ref | **3.39 (1.01-11.35)** | **0.047** |
| Abbreviation: CI, confidence interval; CVD, cardiovascular disease; HR, hazard ratio; NO_2_, nitrogen dioxide; PM_2.5_, particulate matter with diameters of ≤2.5 µm; PRS, polygenic risk score | | | | | | | |
| a. Extreme agers were defined as brain age gap z-score <-2 or > 2 | | | | | | | |
| b. Model 1: chronological age and sex | | | | | | | |
| c. Model 2: model 1 + race, education levels, Townsend deprivation index, body mass index, smoking status, moderate alcohol consumption, regular physical activity, healthy sleep patterns, healthy diet, social isolation, depression, hearing problems, hypertension, type 2 diabetes, high cholesterol, traumatic brain injury, CVD history, NO_2_, and PM_2.5_ | | | | | | | |
| d. Model 3: model 2 + the number of *APOE* ε4 alleles | | | | | | | |
| e. Model 4: model 2 + PRS for Alzheimer's disease | | | | | | | |

| **Supplemental Table 8. The adjusted hazard ratio of dementia by brain age gap z-score and extreme agers^a^ after excluding participants with missing covariates** | | | | | | | |
| --- | --- | --- | --- | --- | --- | --- | --- |
| **Outcomes** | **Per unit increment of z-score** | | **Extreme agers** | | | | |
|  |  |  | **Extreme young** | | **Non-extreme** | **Extreme old** | |
|  | **HR (95% CI)** | **P-value** | **HR (95% CI)** | **P-value** |  | **HR (95% CI)** | **P-value** |
| **All-cause dementia** |  |  |  |  |  |  |  |
| Model 1^b^ | **1.78 (1.66-1.91)** | **<0.001** | **0.37 (0.22-0.65)** | **<0.001** | ref | **4.44 (2.92-6.74)** | **<0.001** |
| Model 2^c^ | **1.69 (1.52-1.87)** | **<0.001** | **0.19 (0.06-0.59)** | **0.004** | ref | **3.05 (1.42-6.55)** | **0.004** |
| Model 3^d^ | **1.66 (1.50-1.84)** | **<0.001** | **0.20 (0.06-0.62)** | **0.005** | ref | **2.79 (1.30-5.99)** | **0.008** |
| Model 4^e^ | **1.67 (1.51-1.86)** | **<0.001** | **0.20 (0.06-0.61)** | **0.005** | ref | **2.86 (1.33-6.14)** | **0.007** |
| **Alzheimer’s disease** |  |  |  |  |  |  |  |
| Model 1 | **1.96 (1.76-2.20)** | **<0.001** | **0.20 (0.07-0.63)** | **0.006** | ref | **5.79 (3.02-11.09)** | **<0.001** |
| Model 2 | **1.92 (1.64-2.26)** | **<0.001** | **-** | 0.959 | ref | **5.72 (2.05-15.90)** | **0.001** |
| Model 3 | **1.89 (1.61-2.23)** | **<0.001** | **-** | 0.958 | ref | **4.72 (1.70-13.12)** | **0.003** |
| Model 4 | **1.92 (1.63-2.26)** | **<0.001** | **-** | 0.960 | ref | **5.11 (1.83-14.22)** | **0.002** |
| **Vascular dementia** |  |  |  |  |  |  |  |
| Model 1 | **2.05 (1.71-2.46)** | **<0.001** | 0.17 (0.02-1.21) | 0.077 | ref | **4.64 (1.43-15.05)** | **0.011** |
| Model 2 | **1.96 (1.47-2.60)** | **<0.001** | - | 0.981 | ref | **9.18 (2.09-40.31)** | **0.003** |
| Model 3 | **1.90 (1.43-2.53)** | **<0.001** | - | 0.981 | ref | **8.07 (1.83-35.62)** | **0.006** |
| Model 4 | **1.90 (1.43-2.53)** | **<0.001** | - | 0.972 | ref | **7.96 (1.79-35.31)** | **0.006** |
| Abbreviation: CI, confidence interval; CVD, cardiovascular disease; HR, hazard ratio; NO_2_, nitrogen dioxide; PM_2.5_, particulate matter with diameters of ≤2.5 µm; PRS, polygenic risk score | | | | | | | |
| a. Extreme agers were defined as brain age gap z-score <-2 or > 2 | | | | | | | |
| b. Model 1: chronological age and sex | | | | | | | |
| c. Model 2: model 1 + race, education levels, Townsend deprivation index, body mass index, smoking status, moderate alcohol consumption, regular physical activity, healthy sleep patterns, healthy diet, social isolation, depression, hearing problems, hypertension, type 2 diabetes, high cholesterol, traumatic brain injury, CVD history, NO_2_, and PM_2.5_ | | | | | | | |
| d. Model 3: model 2 + the number of *APOE* ε4 alleles | | | | | | | |
| e. Model 4: model 2 + PRS for Alzheimer's disease | | | | | | | |

| **Supplemental Table 9. The hazard ratio of dementia by brain age gap z-score and groups of extreme agers^a^ additionally adjusting for vision problems** | | | | | | | |
| --- | --- | --- | --- | --- | --- | --- | --- |
| **Outcomes** | **Per unit increment of z-score** | | **Extreme agers** | | | | |
|  |  |  | **Extreme young** | | **Non-extreme** | **Extreme old** | |
|  | **HR (95% CI)** | **P-value** | **HR (95% CI)** | **P-value** |  | **HR (95% CI)** | **P-value** |
| **All-cause dementia** |  |  |  |  |  |  |  |
| Model 1^b^ | **1.78 (1.58-2.01)** | **<0.001** | **0.35 (0.13-0.94)** | **0.037** | ref | **6.26 (3.42-11.45)** | **<0.001** |
| Model 2^c^ | **1.65 (1.46-1.86)** | **<0.001** | 0.42 (0.16-1.12) | 0.083 | ref | **4.79 (2.59-8.84)** | **<0.001** |
| Model 3^d^ | **1.63 (1.44-1.84)** | **<0.001** | 0.45 (0.17-1.21) | 0.113 | ref | **4.34 (2.35-8.02)** | **<0.001** |
| Model 4^e^ | **1.66 (1.47-1.87)** | **<0.001** | 0.43 (0.16-1.15) | 0.091 | ref | **4.88 (2.64-9.01)** | **<0.001** |
| **Alzheimer’s disease** |  |  |  |  |  |  |  |
| Model 1 | **1.84 (1.51-2.23)** | **<0.001** | 0.20 (0.03-1.46) | 0.113 | ref | **6.24 (1.90-20.42)** | **0.003** |
| Model 2 | **1.77 (1.46-2.15)** | **<0.001** | 0.24 (0.03-1.71) | 0.155 | ref | **5.43 (1.63-18.07)** | **0.006** |
| Model 3 | **1.73 (1.43-2.11)** | **<0.001** | 0.27 (0.04-1.91) | 0.189 | ref | **4.67 (1.40-15.63)** | **0.012** |
| Model 4 | **1.79 (1.47-2.17)** | **<0.001** | 0.25 (0.03-1.77) | 0.164 | ref | **5.74 (1.73-19.11)** | **0.004** |
| **Vascular dementia** |  |  |  |  |  |  |  |
| Model 1 | **2.12 (1.52-2.95)** | **<0.001** | 0.59 (0.08-4.24) | 0.599 | ref | 4.29 (0.56-33.15) | 0.162 |
| Model 2 | **1.82 (1.31-2.53)** | **<0.001** | 0.87 (0.12-6.33) | 0.889 | ref | 2.05 (0.26-16.19) | 0.498 |
| Model 3 | **1.79 (1.29-2.49)** | **<0.001** | 0.96 (0.13-7.05) | 0.971 | ref | 2.03 (0.26-16.10) | 0.501 |
| Model 4 | **1.82 (1.31-2.52)** | **<0.001** | 0.91 (0.12-6.69) | 0.925 | ref | 2.00 (0.25-15.92) | 0.511 |
| Abbreviation: CI, confidence interval; CVD, cardiovascular disease; HR, hazard ratio; NO_2_, nitrogen dioxide; PM_2.5_, particulate matter with diameters of ≤2.5 µm; PRS, polygenic risk score | | | | | | | |
| a. Extreme agers were defined as brain age gap z-score <-2 or > 2 | | | | | | | |
| b. Model 1: chronological age and sex | | | | | | | |
| c. Model 2: model 1 + race, education levels, Townsend deprivation index, body mass index, smoking status, moderate alcohol consumption, regular physical activity, healthy sleep patterns, healthy diet, social isolation, depression, hearing problems, hypertension, type 2 diabetes, high cholesterol, traumatic brain injury, CVD history, NO_2_, and PM_2.5_, and vision problems | | | | | | | |
| d. Model 3: model 2 + the number of *APOE* ε4 alleles | | | | | | | |
| e. Model 4: model 2 + PRS for Alzheimer's disease | | | | | | | |

| **Supplemental Table 10. The adjusted hazard ratio of dementia by brain age gap z-score and groups of extreme agers^a^ among participants** **≥ 60 years old** | | | | | | | |
| --- | --- | --- | --- | --- | --- | --- | --- |
| **Outcomes** | **Per unit increment of z-score** | | **Extreme agers** | | | | |
|  |  |  | **Extreme young** | | **Non-extreme** | **Extreme old** | |
|  | **HR (95% CI)** | **P-value** | **HR (95% CI)** | **P-value** |  | **HR (95% CI)** | **P-value** |
| **All-cause dementia** |  |  |  |  |  |  |  |
| Model 1^b^ | **1.73 (1.59-1.88)** | **<0.001** | **0.45 (0.26-0.78)** | **0.005** | ref | **4.52 (1.87-10.93)** | **0.001** |
| Model 2^c^ | **1.66 (1.53-1.80)** | **<0.001** | **0.49 (0.28-0.84)** | **0.01** | ref | **4.41 (1.82-10.7)** | **0.001** |
| Model 3^d^ | **1.62 (1.49-1.75)** | **<0.001** | **0.51 (0.30-0.89)** | **0.017** | ref | **3.65 (1.50-8.85)** | **0.004** |
| Model 4^e^ | **1.63 (1.50-1.77)** | **<0.001** | **0.49 (0.29-0.85)** | **0.012** | ref | **3.85 (1.59-9.36)** | **0.003** |
| **Alzheimer’s disease** |  |  |  |  |  |  |  |
| Model 1 | **1.92 (1.70-2.18)** | **<0.001** | **0.23 (0.08-0.73)** | **0.012** | ref | **4.89 (1.21-19.76)** | **0.026** |
| Model 2 | **1.87 (1.65-2.13)** | **<0.001** | **0.25 (0.08-0.77)** | **0.016** | ref | **4.64 (1.14-18.83)** | **0.032** |
| Model 3 | **1.79 (1.58-2.04)** | **<0.001** | **0.26 (0.08-0.82)** | **0.022** | ref | **3.55 (0.87-14.45)** | **0.076** |
| Model 4 | **1.83 (1.61-2.07)** | **<0.001** | **0.25 (0.08-0.78)** | **0.017** | ref | **4.05 (0.99-16.46)** | **0.051** |
| **Vascular dementia** |  |  |  |  |  |  |  |
| Model 1 | **2.14 (1.75-2.62)** | **<0.001** | 0.19 (0.03-1.37) | 0.099 | ref | **10.08 (2.46-41.29)** | **0.001** |
| Model 2 | **2.02 (1.65-2.48)** | **<0.001** | 0.21 (0.03-1.52) | 0.123 | ref | **9.04 (2.19-37.41)** | **0.002** |
| Model 3 | **1.96 (1.60-2.40)** | **<0.001** | 0.23 (0.03-1.62) | 0.140 | ref | **7.89 (1.90-32.69)** | **0.004** |
| Model 4 | **1.96 (1.60-2.41)** | **<0.001** | 0.22 (0.03-1.54) | 0.126 | ref | **7.69 (1.85-31.95)** | **0.005** |
| Abbreviation: CI, confidence interval; CVD, cardiovascular disease; HR, hazard ratio; NO_2_, nitrogen dioxide; PM_2.5_, particulate matter with diameters of ≤2.5 µm; PRS, polygenic risk score | | | | | | | |
| a. Extreme agers were defined as brain age gap z-score <-2 or > 2 | | | | | | | |
| b. Model 1: chronological age and sex | | | | | | | |
| c. Model 2: model 1 + race, education levels, Townsend deprivation index, body mass index, smoking status, moderate alcohol consumption, regular physical activity, healthy sleep patterns, healthy diet, social isolation, depression, hearing problems, hypertension, type 2 diabetes, high cholesterol, traumatic brain injury, CVD history, NO_2_, and PM_2.5_ | | | | | | | |
| d. Model 3: model 2 + the number of *APOE* ε4 alleles | | | | | | | |
| e. Model 4: model 2 + PRS for Alzheimer's disease | | | | | | | |

| **Supplemental Table 11. The adjusted hazard ratio of dementia by brain age gap z-score and groups of extreme agers^a^ excluding first 5-year follow-up** | | | | | | | |
| --- | --- | --- | --- | --- | --- | --- | --- |
| **Outcomes** | **Per unit increment of z-score** | | **Extreme agers** | | | | |
|  |  |  | **Extreme young** | | **Non-extreme** | **Extreme old** | |
|  | **HR (95% CI)** | **P-value** | **HR (95% CI)** | **P-value** |  | **HR (95% CI)** | **P-value** |
| **All-cause dementia** |  |  |  |  |  |  |  |
| Model 1^b^ | **1.69 (1.57-1.82)** | **<0.001** | **0.44 (0.25-0.75)** | **0.003** | ref | **3.40 (2.01-5.74)** | **<0.001** |
| Model 2^c^ | **1.62 (1.50-1.75)** | **<0.001** | **0.47 (0.27-0.82)** | **0.007** | ref | **2.91 (1.72-4.91)** | **<0.001** |
| Model 3^d^ | **1.59 (1.48-1.72)** | **<0.001** | **0.49 (0.28-0.85)** | **0.011** | ref | **2.64 (1.56-4.46)** | **<0.001** |
| Model 4^e^ | **1.60 (1.49-1.73)** | **<0.001** | **0.47 (0.27-0.82)** | **0.008** | ref | **2.86 (1.69-4.84)** | **<0.001** |
| **Alzheimer’s disease** |  |  |  |  |  |  |  |
| Model 1 | **1.85 (1.64-2.08)** | **<0.001** | **0.24 (0.08-0.73)** | **0.013** | ref | **4.89 (2.26-10.57)** | **<0.001** |
| Model 2 | **1.80 (1.59-2.03)** | **<0.001** | **0.25 (0.08-0.78)** | **0.017** | ref | **4.45 (2.05-9.65)** | **<0.001** |
| Model 3 | **1.75 (1.55-1.97)** | **<0.001** | **0.26 (0.08-0.80)** | **0.019** | ref | **3.76 (1.74-8.15)** | **0.001** |
| Model 4 | **1.77 (1.57-2.00)** | **<0.001** | **0.25 (0.08-0.77)** | **0.016** | ref | **4.28 (1.98-9.28)** | **<0.001** |
| **Vascular dementia** |  |  |  |  |  |  |  |
| Model 1 | **2.02 (1.66-2.47)** | **<0.001** | 0.20 (0.03-1.43) | 0.109 | ref | **6.04 (1.85-19.77)** | **0.003** |
| Model 2 | **1.91 (1.56-2.33)** | **<0.001** | 0.22 (0.03-1.59) | 0.134 | ref | **5.24 (1.59-17.24)** | **0.006** |
| Model 3 | **1.86 (1.52-2.27)** | **<0.001** | 0.24 (0.03-1.70) | 0.152 | ref | **4.74 (1.44-15.58)** | **0.010** |
| Model 4 | **1.87 (1.53-2.28)** | **<0.001** | 0.23 (0.03-1.62) | 0.138 | ref | **4.98 (1.52-16.38)** | **0.008** |
| Abbreviation: CI, confidence interval; CVD, cardiovascular disease; HR, hazard ratio; NO_2_, nitrogen dioxide; PM_2.5_, particulate matter with diameters of ≤2.5 µm; PRS, polygenic risk score | | | | | | | |
| a. Extreme agers were defined as brain age gap z-score <-2 or > 2 | | | | | | | |
| b. Model 1: chronological age and sex | | | | | | | |
| c. Model 2: model 1 + race, education levels, Townsend deprivation index, body mass index, smoking status, moderate alcohol consumption, regular physical activity, healthy sleep patterns, healthy diet, social isolation, depression, hearing problems, hypertension, type 2 diabetes, high cholesterol, traumatic brain injury, CVD history, NO_2_, and PM_2.5_ | | | | | | | |
| d. Model 3: model 2 + the number of *APOE* ε4 alleles | | | | | | | |
| e. Model 4: model 2 + PRS for Alzheimer's disease | | | | | | | |

**Supplemental Figures**


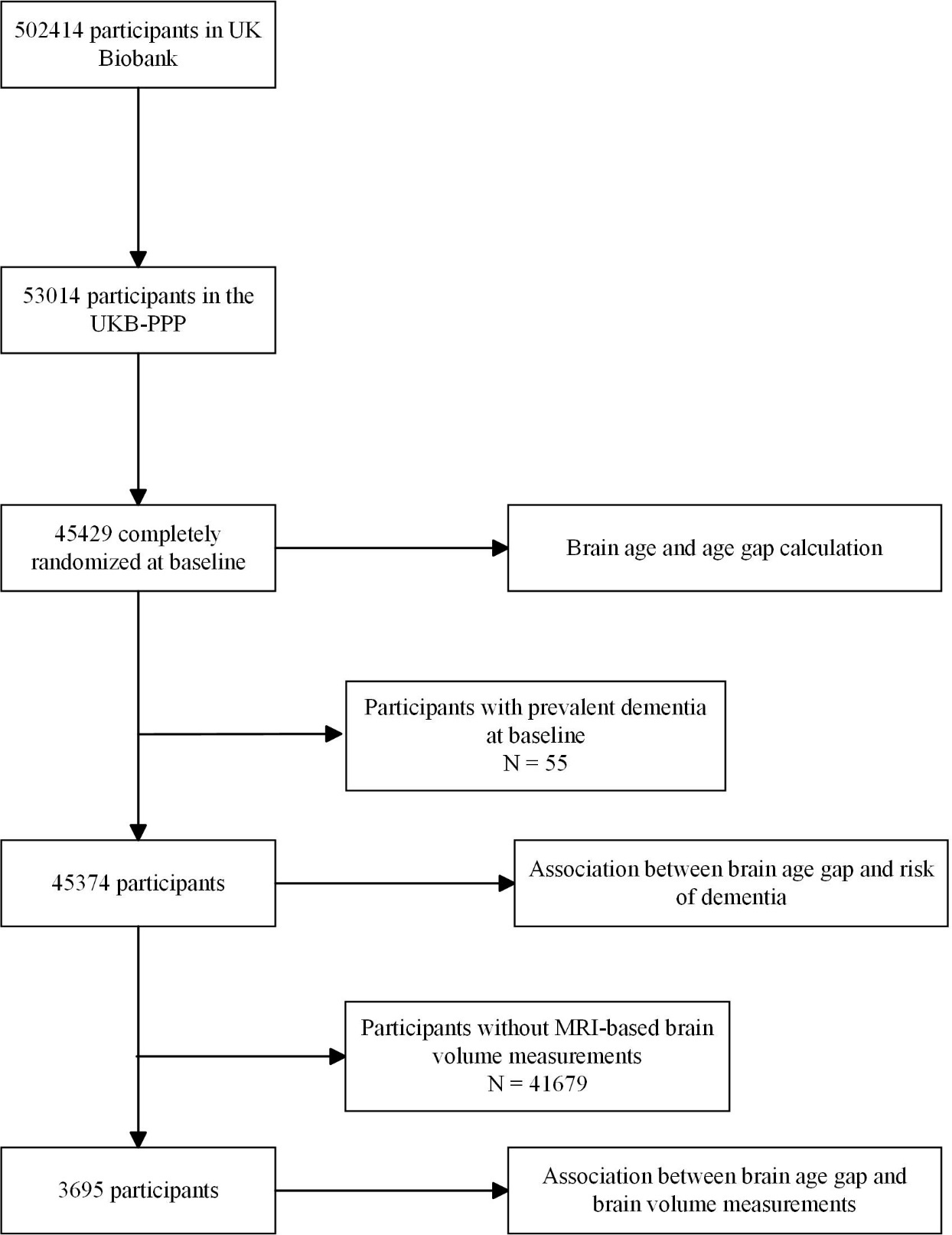


Supplemental Figure 1. Flowchart of the study


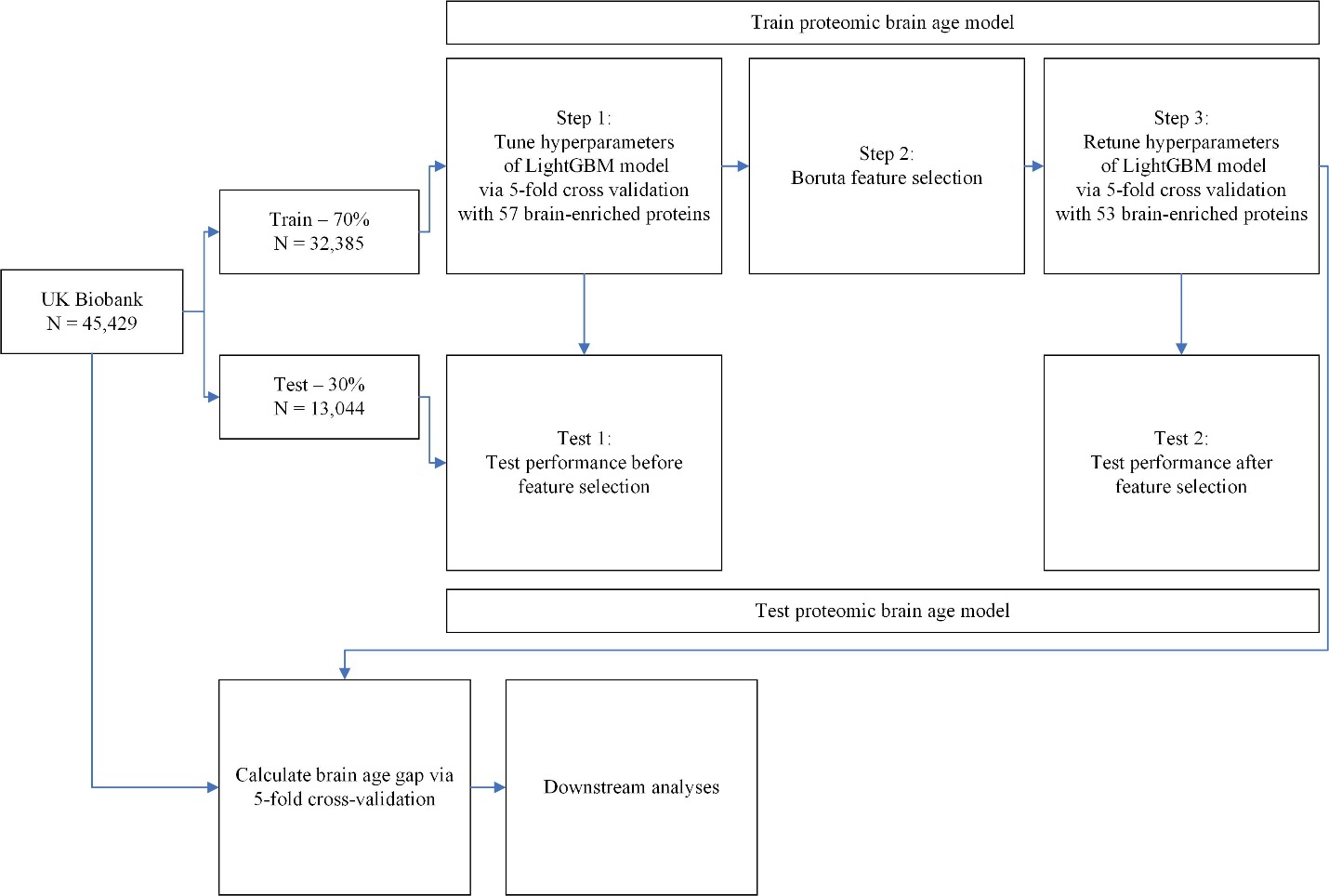


Supplemental Figure 2. Overview of proteomic brain age model training and testing, and brain age calculation


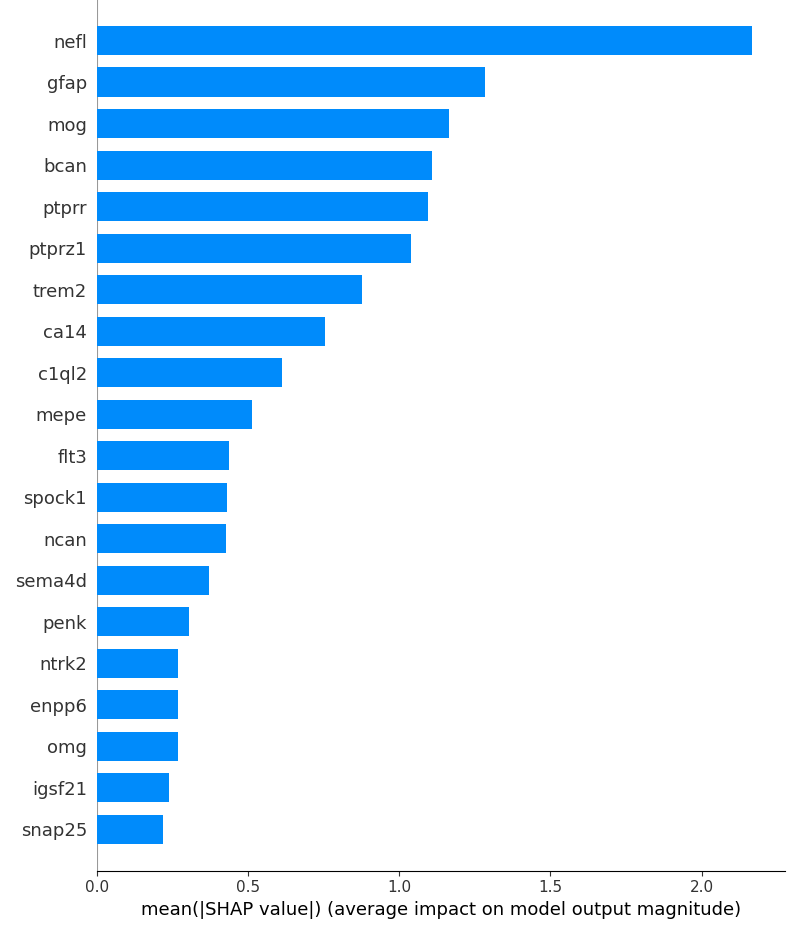


Supplemental Figure 3. Twenty proteins with largest SHAP values in proteomic age prediction model


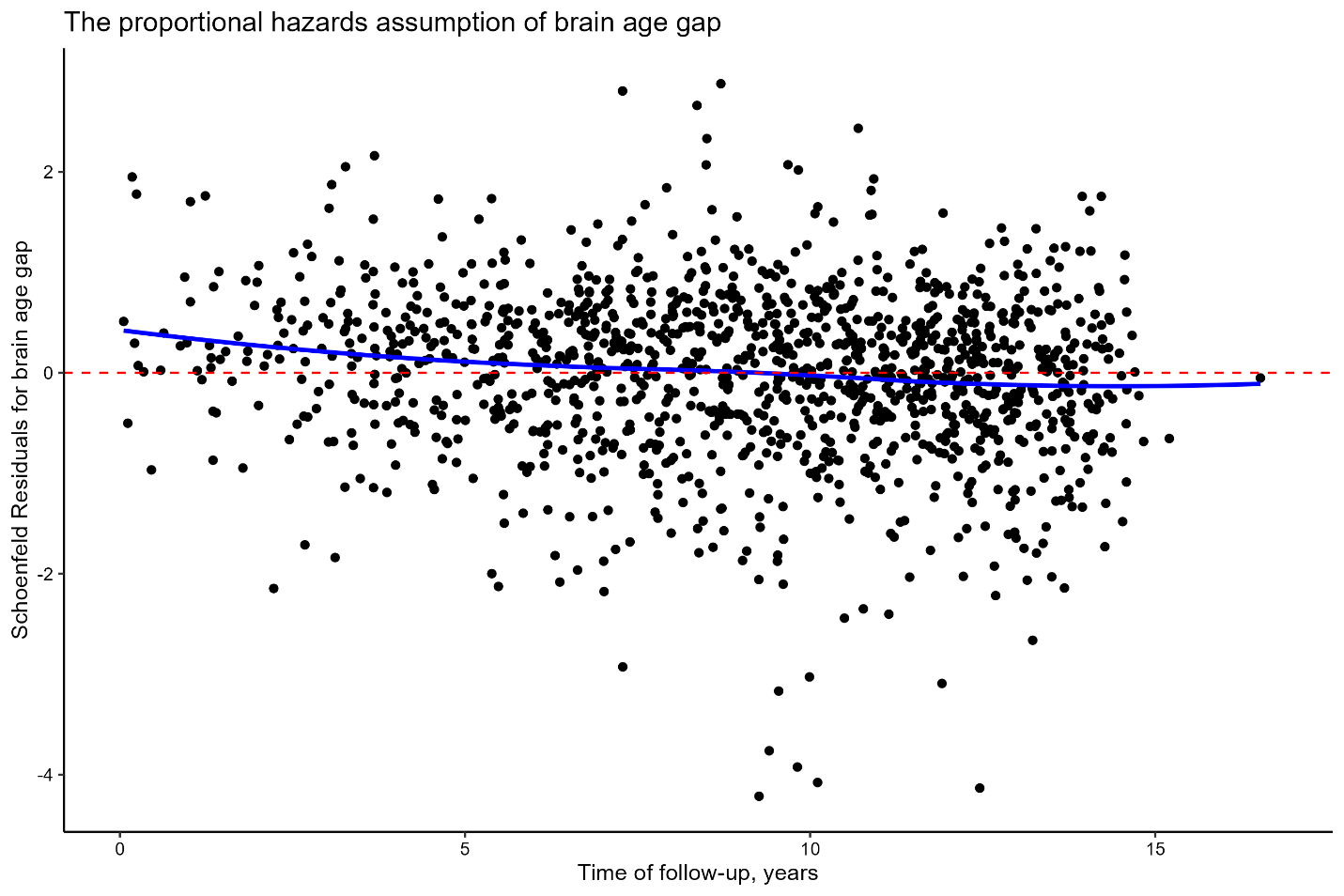


Supplemental Figure 4. Schoenfeld residuals by brain age gap over time. The model adjusted for chronological age, sex, race, education levels, Townsend deprivation index, body mass index, smoking status, alcohol consumption, physical activity, healthy sleep patterns, healthy diet, social isolation, depression, hearing problems, hypertension, type 2 diabetes, high cholesterol, traumatic brain injury, history of CVD, NO_2_, PM_2.5_.


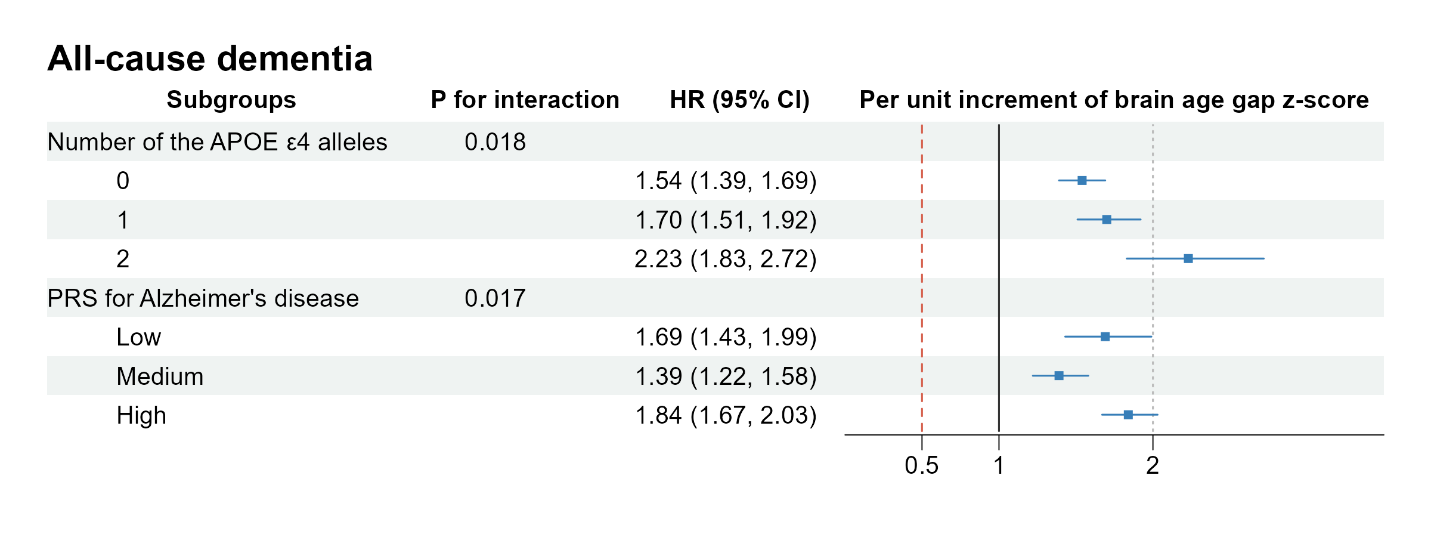

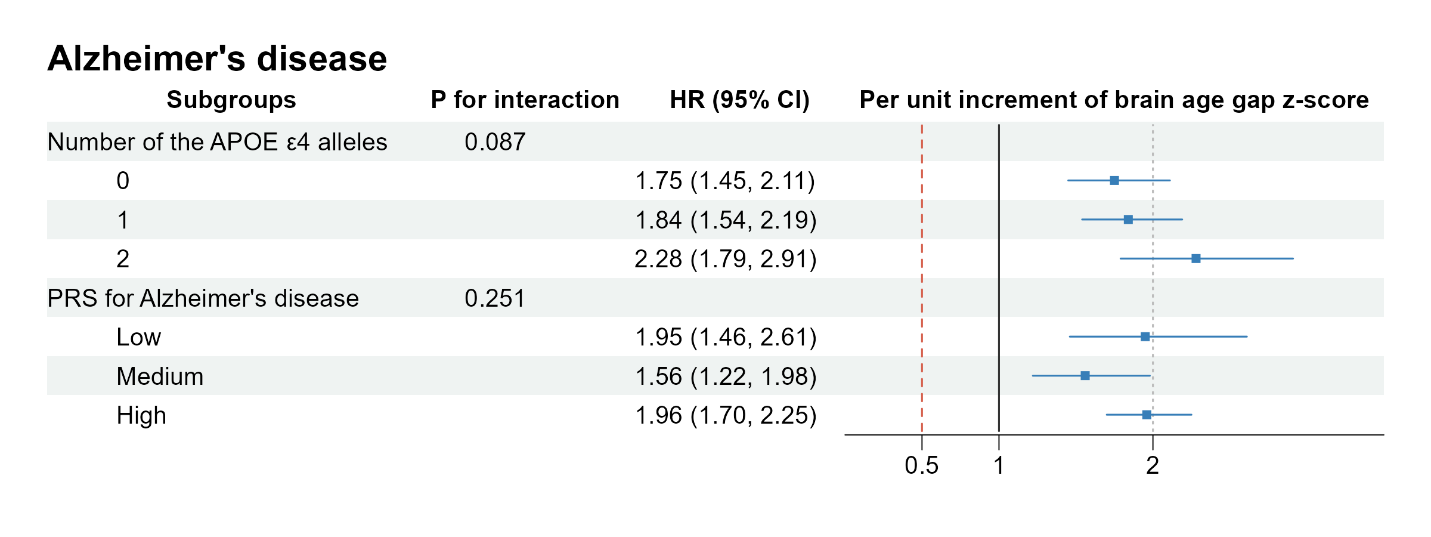

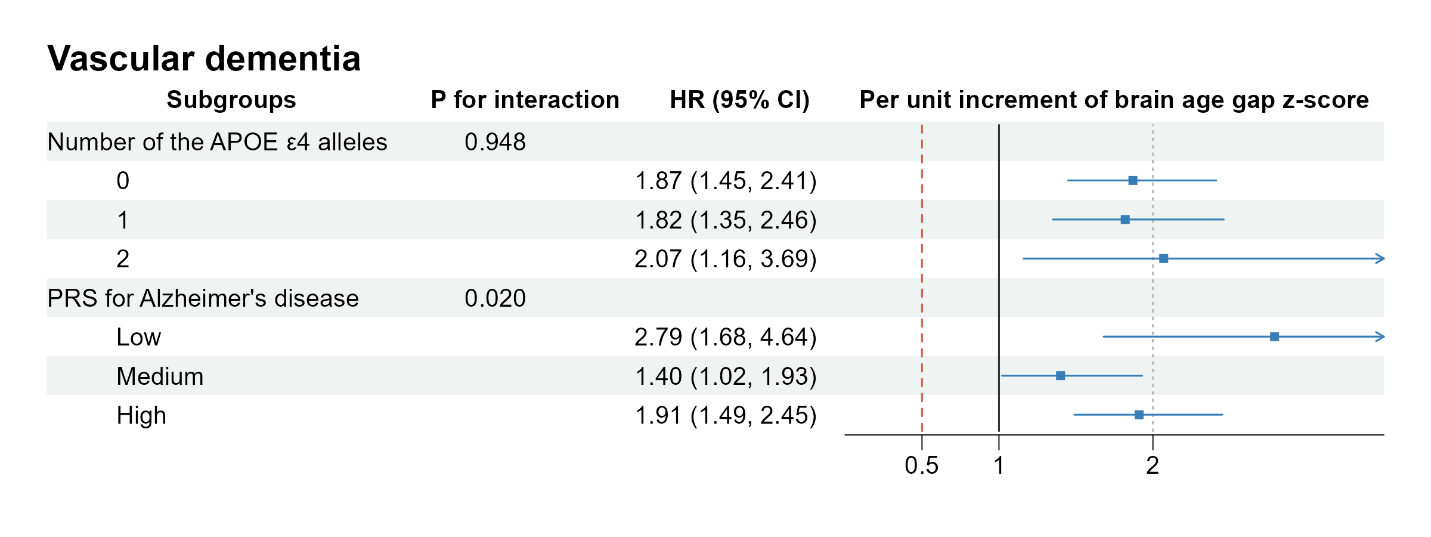


Supplemental Figure 5. Adjusted hazard ratio of dementia for per-unit increment of brain age gap z-score by subgroups of genetic susceptibility. The model adjusted for chronological age, sex, race, education levels, Townsend deprivation index, body mass index, smoking status, alcohol consumption, physical activity, healthy sleep patterns, healthy diet, social isolation, depression, hearing problems, hypertension, type 2 diabetes, high cholesterol, traumatic brain injury, history of CVD, NO_2_, PM_2.5_, and the number of *APOE* ε4 alleles.

**Reference**

1. Sun BB, Chiou J, Traylor M, et al. Plasma proteomic associations with genetics and health in the UK Biobank. *Nature*. 2023/10/01 2023;622(7982):329-338. doi:10.1038/s41586-023-06592-6

2. The GTEx Consortium atlas of genetic regulatory effects across human tissues. *Science (New York, NY)*. Sep 11 2020;369(6509):1318-1330. doi:10.1126/science.aaz1776

3. Love MI, Huber W, Anders S. Moderated estimation of fold change and dispersion for RNA-seq data with DESeq2. *Genome biology*. 2014;15(12):550. doi:10.1186/s13059-014-0550-8

4. Uhlén M, Fagerberg L, Hallström BM, et al. Proteomics. Tissue-based map of the human proteome. *Science (New York, NY)*. Jan 23 2015;347(6220):1260419. doi:10.1126/science.1260419

5. Oh HS, Rutledge J, Nachun D, et al. Organ aging signatures in the plasma proteome track health and disease. *Nature*. Dec 2023;624(7990):164-172. doi:10.1038/s41586-023-06802-1

6. Lundberg SM, Erion G, Chen H, et al. From Local Explanations to Global Understanding with Explainable AI for Trees. *Nat Mach Intell*. Jan 2020;2(1):56-67. doi:10.1038/s42256-019-0138-9

7. Sudlow C, Gallacher J, Allen N, et al. UK Biobank: An Open Access Resource for Identifying the Causes of a Wide Range of Complex Diseases of Middle and Old Age. *PLoS medicine*. 2015;12(3):e1001779. doi:10.1371/journal.pmed.1001779

8. Carter AR, Gill D, Davies NM, et al. Understanding the consequences of education inequality on cardiovascular disease: mendelian randomisation study. *BMJ*. 2019;365:l1855. doi:10.1136/bmj.l1855

9. Lourida I, Hannon E, Littlejohns TJ, et al. Association of Lifestyle and Genetic Risk With Incidence of Dementia. *Jama*. Aug 6 2019;322(5):430-437. doi:10.1001/jama.2019.9879

10. Services UDoHaH. 2015-2020 Dietary guidelines for Americans. Accessed 01/03, 2024. <https://health.gov/sites/default/files/2019-09/2015-2020_Dietary_Guidelines.pdf>

11. Piercy KL, Troiano RP, Ballard RM, et al. The Physical Activity Guidelines for Americans. *Jama*. Nov 20 2018;320(19):2020-2028. doi:10.1001/jama.2018.14854

12. Mozaffarian D. Dietary and Policy Priorities for Cardiovascular Disease, Diabetes, and Obesity: A Comprehensive Review. *Circulation*. Jan 12 2016;133(2):187-225. doi:10.1161/circulationaha.115.018585

13. Fan M, Sun D, Zhou T, et al. Sleep patterns, genetic susceptibility, and incident cardiovascular disease: a prospective study of 385 292 UK biobank participants. *European heart journal*. Mar 14 2020;41(11):1182-1189. doi:10.1093/eurheartj/ehz849

14. Elovainio M, Hakulinen C, Pulkki-Råback L, et al. Contribution of risk factors to excess mortality in isolated and lonely individuals: an analysis of data from the UK Biobank cohort study. *The Lancet Public health*. Jun 2017;2(6):e260-e266. doi:10.1016/s2468-2667(17)30075-0

15. Kloog I, Sorek-Hamer M, Lyapustin A, et al. Estimating daily PM(2.5) and PM(10) across the complex geo-climate region of Israel using MAIAC satellite-based AOD data. *Atmos Environ (1994)*. Dec 2015;122:409-416. doi:10.1016/j.atmosenv.2015.10.004

16. Eeftens M, Beelen R, de Hoogh K, et al. Development of Land Use Regression models for PM(2.5), PM(2.5) absorbance, PM(10) and PM(coarse) in 20 European study areas; results of the ESCAPE project. *Environ Sci Technol*. Oct 16 2012;46(20):11195-205. doi:10.1021/es301948k
